# From Feedstock to Future Chemicals: Rethinking Carbon Sources in Industrial Propylene Clusters

**DOI:** 10.1021/acssuschemeng.5c05287

**Published:** 2025-10-13

**Authors:** Inna Stepchuk, Mar Pérez-Fortes, Andrea Ramírez

**Affiliations:** † Department of Engineering Systems and Services, Faculty of Technology, Policy and Management, 2860 Delft University of Technology, Jaffalaan 5, Delft 2628 BX, The Netherlands; ‡ Department of Chemical Engineering, Faculty of Applied Sciences, Delft University of Technology, Van der Maasweg 9, Delft 2628 CN, The Netherlands

**Keywords:** industrial defossilization, techno-economic assessment and environmental assessment (TEE assessment), petrochemical cluster, methyl-*tert*-butyl ether (MTBE), propylene glycol (PG), polyol, propylene oxide (PO), renewable carbon

## Abstract

The rising pressure to defossilize the chemical industry has driven research toward producing chemicals that use alternative carbon sources (ACS). However, the challenges and impacts of replacing already implemented processes and symbiotic relationships remain largely underexplored. This paper systematically assesses the impacts of defossilizing existing processes, both individually and simultaneously, in a propylene cluster in the Port of Rotterdam, the Netherlands. Nine fossil-based processes and three ACS-based processes (i.e., CO_2_-based polyol, biopropylene glycol (bio-PG), and biomethyl-tert-butyl-ether (bio-MTBE)) were included in the assessment. Integrating a single ACS-based process enlarges the propylene cluster and results in an excess of upstream chemicals that are no longer required by the ACS processes. Still, relatively simple technologies can reduce total energy and water use, resulting in lower direct CO_2_ emissions and water consumption of the cluster. Deploying multiple processes in parallel can drive the full defossilization of the cluster, but it requires a complete overhaul. The results illustrate the extent to which combining ACS-based processes could change the layout of an existing petrochemical cluster, affecting its performance. The paper stresses the importance of assessing such deployments, considering the existing conditions in industrial clusters.

## Graphical abstract

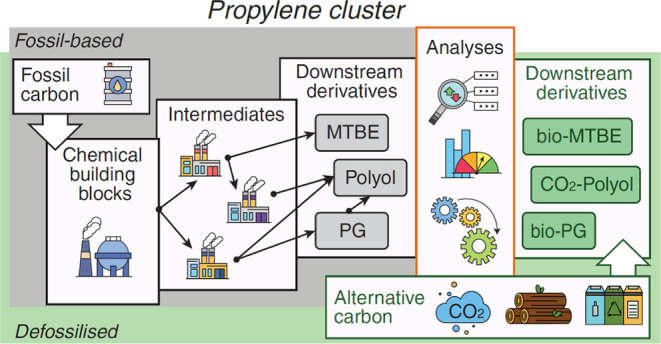

## 1 Introduction

Propylene is an important chemical building block (CBB) for most petrochemical clusters, such as the Port of Rotterdam (PoR) in the Netherlands. Numerous downstream derivatives (DD) are produced from propylene, such as propylene glycol (PG), polyol, propylene glycol methyl ether (PGME), polypropylene, methyl-tert-butyl ether (MTBE), isopropyl alcohol, styrene or acetone (e.g., see Figure 1). These chemicals are widely used in industrial and consumer applications as solvents, additives for paints, gasoline, packaging or deicing agents.^1^ Although most DD are still produced from fossil-based feedstocks, there is a growing interest in defossilizing their production by utilizing alternative carbon sources (ACS), such as biomass, waste, and carbon dioxide (CO_2_).^2^

**Figure 1:**
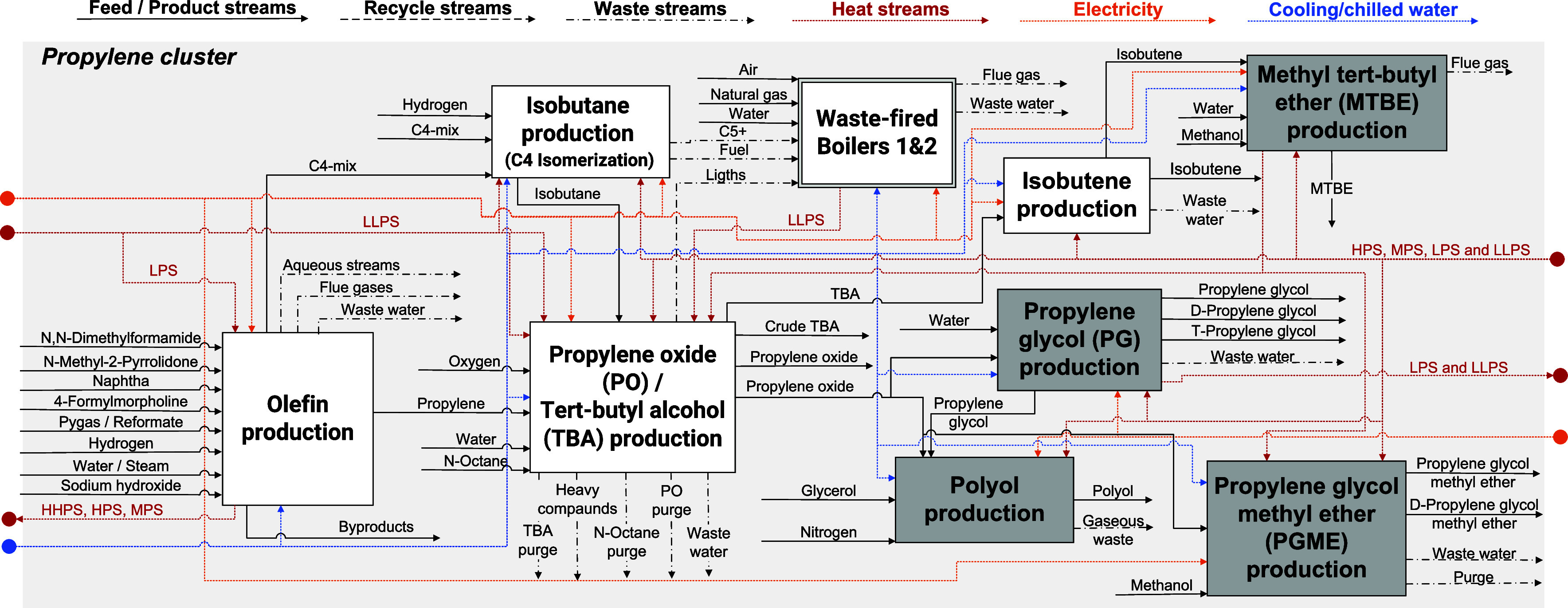
Block flow diagram for the current fossil-based propylene cluster at the Port of Rotterdam. The DD production processes are depicted in gray. LLPS – very low-pressure steam; LPS – low-pressure steam; MPS – medium-pressure steam; HPS – high-pressure steam.

Achieving the complete defossilization of downstream chemicals is a significant challenge. Figure 2 provides an overview of the key ACS-based pathways for the production of polyol, PG, PGME and MTBE found in the literature. Further details, including references used, are reported in the Tables S1–S3. Currently, many of them only enable partial defossilization of DD production. For instance, ACS-based polyols can be produced by copolymerization of propylene oxide (PO) with CO_2_.^3,4^ Similarly, using ACS-based methanol in PGME can reduce fossil carbon input by only 25%, with the remaining still produced from fossil sources. In both cases, achieving full defossilization requires the use of nonfossil PO. Although PO can theoretically be produced from ACS,^5,6^ no direct synthesis routes have been reported in the literature (see Figure 2). The same limitation applies to other chemicals, such as PG and MTBE, which can be produced indirectly from glycerol^7^ and biobased isobutene (bio-IBN).^8^ These indirect routes allow for complete substitution of fossil-based carbon in the DD production using ACS-based feedstocks. While process-level defossilization is technically feasible, the impacts of defossilizing DD at the cluster level remain largely unexplored.

**Figure 2:**
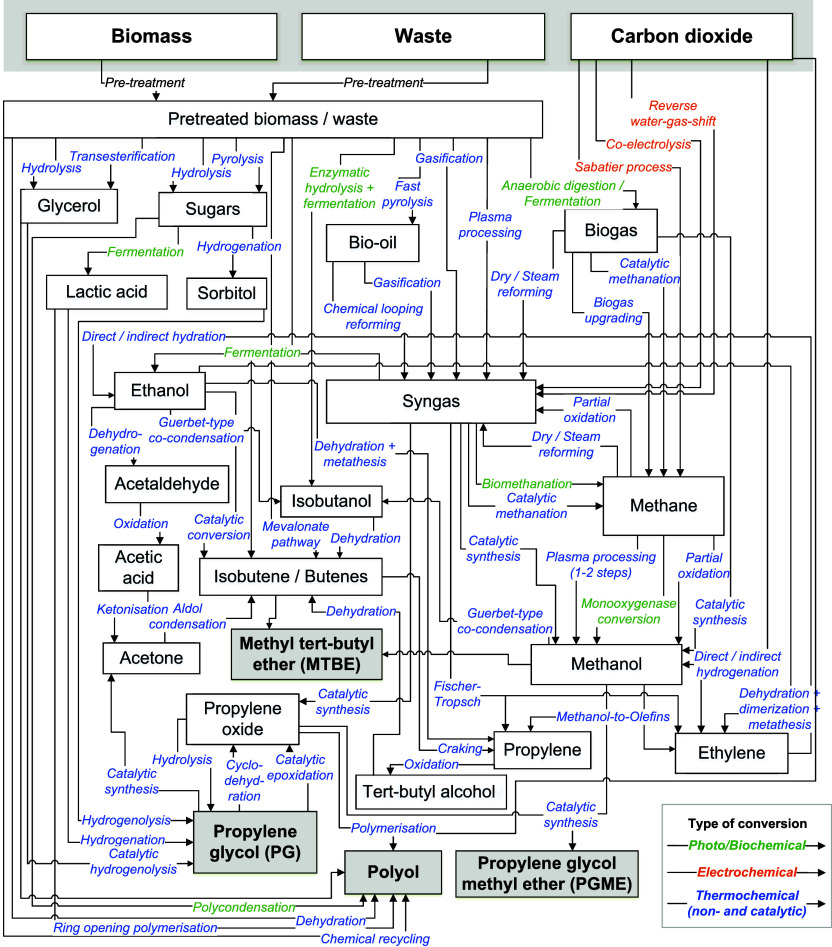
Overview of key ACS-based production pathways for polyol, PG, PGME and MTBE based on the literature. The list of references for the routes can be found in the Tables S1 to −S3. In color, the type of conversions are indicated. Reproduced or adapted with permission from.^8^ Copyright 2025 Elsevier Ltd.

Industrial clusters are complex systems with highly interconnected mass and energy flows, and they often face constraints such as limited land availability for new processes. This complexity is evident in the propylene cluster of PoR (see Figure 1), where multiple value chains of downstream derivatives rely on shared upstream chemicals. For example, chemical building blocks such as propylene and isobutane are used to produce intermediate chemicals like PO and TBA.^9^ The intermediates are then utilized in the production of DD, such as polyol, PGE, PGME and MTBE.^2^ Although ACS-based routes for DD production may also share common upstream chemicals, they typically differ from those used in fossil-based routes (see Figure 2). However, these differences in production routes can create opportunities for DD to be used in circular integration in the cluster, such as for producing intermediate chemicals. For example, bio-PG can be further converted into PO,^10^ which can be employed to defossilize CO_2_-based polyol and PGME production (see Figure 2).

However, integrating these alternative routes could significantly change the cluster’s boundaries and affect its overall performance. Our previous study^8^ examined the defossilization of MTBE production by introducing a bio-IBN process. This case study highlighted the significant impacts of even a single DD process on an existing cluster. To the best of the authors’ knowledge, a scenario considering the deployment of multiple alternative DD processes within an existing industrial cluster remains highly unexplored in the literature.

This paper aims to systematically assess the cascading impacts of integrating multiple ACS-based processes for DD production in existing petrochemical clusters. Specifically, it examines the impacts of deploying ACS-based processes into the existing structure of the propylene cluster at the Port of Rotterdam (PoR), the Netherlands. The aim of this paper is twofold: first, to assess and compare ACS processes to their fossil-based counterparts; and second, to evaluate techno-economic performance and implications in the direct CO_2_ emissions and water use of integrating ACS-based DD processes within an existing industrial cluster, both individually and simultaneously.

## 2 Materials and Methods

This work builds upon and extends a previous approach to assess the defossilization impacts of deploying ACS technologies in an existing industrial cluster.^8^ It consists of four main steps: (i) defining the scope and system boundaries, (ii) process modeling, (iii) assessing performance at the process level, and (iv) assessing impacts at the cluster level.

### 2.1 Scope Definition and System Boundaries

The approach focuses on defossilizing the production of three downstream derivatives, polyol, PG and MTBE, shown in Figure 1 (gray boxes). The study uses an in-house model developed at TU Delft of the industrial cluster in the PoR. The model includes mass and energy interconnections between processes within the petrochemical cluster, mimicking those in the PoR. For more details, see.^11^ For the assessment, two system boundaries were defined: (i) “process level” assessing the stand-alone production processes; and (ii) “cluster level”, including upstream and downstream production processes involved in the cluster. Note that the processes (white and gray boxes in Figure 1) belong to different companies in the cluster.

All required feedstocks and chemicals were assumed to be bought from the market, preferably from ACS-based origin, such as ethanol, methanol, and glycerol. Byproducts not used in downstream processes were assumed to be sold. Mimicking the current situation in the Port of Rotterdam, utilities (steam, electricity) were assumed to be generated and reused internally within the DD processes. When this was not feasible, utilities were either purchased from or sold to the market. These utilities were assumed to be fossil-based in line with current market conditions. While full heat integration was outside the scope of this paper, key heat exchanges were identified using pinch point analysis in Aspen Energy Analyzer to minimize external utility demand. Flue gases were assumed to be treated before release into the atmosphere. Hazardous and liquid waste were assumed to be treated off-site, with associated costs included in operational expenditures. Byproducts were assumed to be directly sent to the next facility or sold, without storage.

### 2.2 Process Modeling

Fossil- and ACS-based processes were modeled in Aspen Plus v12, assuming continuous operation for 8000 h per year. The capacities and product purities of the ACS-based processes were set equal to those of the fossil-based counterparts in the PoR. Detailed descriptions of the process flow diagrams, modeling assumptions and property methods used, and validation of model outputs, such as mass and energy balances, are provided in the Table S4. Due to the limited data availability for the whole ACS-based processes, validation was performed at the level of individual units rather than for the entire ACS-based process.

### 2.3 Assessment of Individual Processes

This study evaluates three ACS-based processes: (i) polyol production by partial substitution of PO by CO_2_ (i.e., CO_2_-based polyol); (ii) PG production from bioglycerol (i.e., bio-PG), and (iii) MTBE production from biomass (i.e., bio-MTBE). For MTBE production, this work uses the results from our previous study on bio-IBN production.^8^ The IBN process, whether fossil- or ACS-based, was considered within the boundaries of the MTBE process.

Both fossil- and ACS-based processes were assessed using the techno-economic and environmental (TEE) indicators. Definitions and equations are provided in the Table S7. The technical performance of each process was assessed based on: carbon feedstock, net heat and power requirements. For the economic evaluation, four indicators were used: capital (CAPEX) and operating (OPEX) expenditures, equivalent annual operating costs (EAOC) and minimum selling price (MSP). Mass, energy balances and bare equipment costs were retrieved from the Aspen Plus models (see Table S4). Market prices for raw materials, utilities and disposal of wastewater and hazardous wastes were obtained from publicly available data (see Table S5). All prices were adjusted to 2018 values using the Chemical Producer Price Index.^12^

The environmental indicators included: water consumption, bare land requirements, and total CO_2_ emissions, including Scope 1 (direct process emissions) and Scope 2 (indirect emissions from energy use). Water consumption was defined as the amount of water permanently withdrawn from the source, including losses from utility usage. The bare land requirement was based on the footprint of the main equipment, such as columns, reactors, and heat exchangers. Scope 2 emissions were calculated using emission factors (see Table S6) derived from the stream compositions in the Aspen Plus models of utility systems, including natural gas and waste-fired boilers and combined-cycle power plants.^11^

### 2.4 Impact Assessment at the Cluster Level

Two case studies were analyzed to evaluate the impact of integrating ACS-based processes. Case study 1: individual defossilization, where ACS-based processes were introduced one at a time. Case study 2: simultaneous defossilization, where multiple ACS-based processes were introduced at the same time. In both cases, the following assumptions were applied: (i) each ACS-based DD process was required to meet the existing market demand currently fulfilled by its fossil-based counterpart; and (ii) upstream processes were included when necessary to meet the existing demand for downstream production. After each integration, the techno-economic and environmental implications were assessed at the cluster level.

#### 2.4.1 Analysis of Structural Changes

To identify structural changes within the cluster, a graphical network was used to represent interconnections between processes, following the approach of.^13^ First, the cases were compared regarding key differences in the processes (nodes) and their connections (mass and energy flows as links). Structural changes in terms of links and nodes were identified and categorized as follows: (i) elements completely removed (100% removal of a node or link), (ii) elements partially affected (partially reduced/added), (iii) elements that remain unchanged (same flow and composition, and processes, as in the fossil fuel-based counterpart), and (iv) new elements are added (100% new). It was assumed that a process could only be removed if its main product was no longer required in the cluster. Otherwise, it was classified as affected.

#### 2.4.2 Techno-Economic and Environmental Implications

The TEE assessment at the cluster level took into account the interconnectivity of units within the fossil- and ACS-based clusters. Therefore, clusters were studied as an array of production processes that share energy and material flows. Since processes within the cluster generate different products, byproducts or utilities, every production process was examined regarding the potential outputs that could be sold to the market and an economic allocation (i.e., per revenue of DD produced) was applied for the assessment (see Table S9). The same TEE indicators were used as for the process level (see Section 2.3). No prices were assigned to chemicals produced and used internally within the cluster (i.e., they were considered directly available for internal use). To minimize variations in the capacities of upstream processes, caused by the differing demands for the same chemicals required for ACS-based DD processes, a 30% flexibility margin in upstream processes was applied. This reflects a typical operational flexibility of chemical processes reported in the literature.^14^

An “ambitious” approach was considered, following the defossilization of the cluster, assuming no longer a market for fossil-based chemicals. Therefore, any fossil-based products and/or byproducts still needed to be produced but not used internally in the cluster were assumed to have a zero value and to be treated as waste, incurring disposal costs (see Table S5). Where possible, material and energy flows from the removed processes were redirected for internal use in the cluster. If reuse was impossible, they were assumed to be sold on the market. If a process was entirely removed, its associated equipment was considered to be sold, with no potential for repurposing in ACS-based processes. The salvage market value (S_V_) of such equipment was calculated assuming a service life of 25 years and a 10% annual depreciation rate (see Table S8).

## 3 Results and Discussion

### 3.1 Process Level

Table 1 presents a summary of the techno-economic and environmental indicators of the fossil- and ACS-based processes. Compared to the fossil-based ones (see Figures S1 and S2), the CO_2_-based polyol process utilizes 38% less PO, resulting in a 40% reduction of fossil-based carbon input. For the bio-PG and bio-MTBE processes, the carbon feedstock is entirely biogenic. These ACS-based processes use glycerol, IBN, and methanol produced from biomass, thereby eliminating fossil-based PO, TBA, and methanol used in the fossil counterpart.

**Table 1:** Techno-Economic and Environmental Indicators for the Assessment of the Stand-Alone Processes (Based on Table S7)­^a^

			Polyol		PG		MTBE	
Indicator	Abbr	Units	Fossil	ACS	Fossil	ACS	Fossil	ACS
***Techno-economic***								
Carbon feedstock	C_f_	kt/y	43	34	38	38	431	895
Net steam consumption	TEC_steam_	PJ/y	0.04	0.01	0.7	0.6	0.6	–2
Net cooling water consumption	TEC_CW_	PJ/y	0.16	0.06	0.7	0.5	0.6	54
Net electricity consumption	TEC_electricity_	PJ/y	-	0.01	0.002	-	0.03	13
Capital expenditures	CAPEX	MEUR	2	22	22	32	31	1,092
Operational expenditures	OPEX	MEUR/y	121	80	128	110	533	642
Equivalent annual operating costs	EAOC	MEUR/y	121	82	145	118	536	744
Minimum selling price	MSP	EUR/kg	1.7	1.2	1.6	1.4	1.3	2.1
***Environmental***								
Total water consumption	TWC	kt/y	55	21	345	217	864	17,593
Total bare land requirement	TL	m^2^	8	15	68	78	84	1,854
Scope 1: Process-related	CO_2_^scope 1^	kt CO_2‑eq/y_	0	0.5	0	0	0	1,583^b^
Scope 2: Energy-related^c^	CO_2_^scope 2^	kt CO_2‑eq/y_	4.6	2.9	138	134	157	2,204
Total CO_2_ emissions	CO_2_^total^	kt CO_2‑eq/y_	4.6	3.4	138	134	157	3,787

a Numbers in the table are allocated (see Table S9).

b Biogenic origin.

c Assuming the current energy mix of the industrial steam production (see Section 2.3).

The energy needs of ACS-based processes differ significantly from their fossil-based counterparts (see Tables S10 and S11). The CO_2_-based polyol process does not require very low-pressure steam (LLPS) and has 2.5 times lower cooling water consumption. However, due to the different pressure levels, the ACS-based process requires more electricity (i.e., for compressors) and utilizes twice as much low-pressure steam (LPS). While the fossil-based PG process operates at 20 bar, the biobased process operates at ambient pressure (1.02 bar). This requires 18% less medium-pressure steam (MPS) and 1.6 times less cooling water. Additionally, the bio-PG process produces 40 times more LPS, fully meeting its needs and exporting the surplus. In comparison to the fossil-based process, the bio-MTBE process consumes significantly more electricity and cooling water, primarily due to the size and complexity of the bio-IBN process; for more details, we refer to^8^). However, the bio-IBN process generates in situ steam, fully covering its own and the MTBE process’s needs for LPS and high-pressure steam (HPS), with excess steam exported to the market.

As shown in Figure 3, the CAPEX of all ACS-based processes are higher than that of their fossil-based counterparts (see Table S4 for details). However, the OPEX of the CO_2_-based polyol and bio-PG processes are significantly lower, by 34% and 20%, respectively. This reduction is primarily due to the partial or complete elimination of PO usage, which has a higher price as a raw material than CO_2_ or glycerol (see Table S5). Despite the reduction in costs of raw materials, the OPEX of the bio-MTBE process is twice as high as the fossil-based process. This is mainly due to the high utility costs (i.e., cooling water and electricity) associated with the bio-IBN production, which accounts for 40% of its total OPEX.

**Figure 3:**
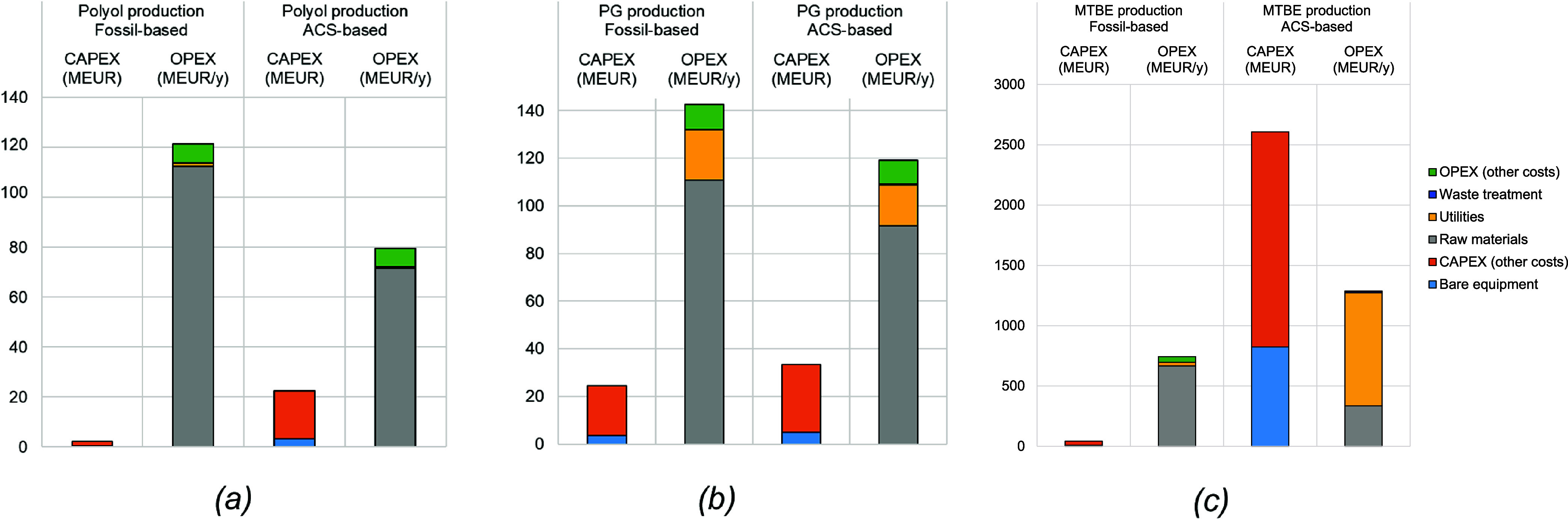
Total CAPEX and OPEX of fossil- and ACS-based (a) polyol, (b) PG and (c) MTBE production processes (for further details, refer to the Table S13).

Note that the fluctuations in the prices of ACS materials and utilities differently affect the OPEX of ACS-based processes (see Table S14). The OPEX of the CO_2_-based polyol process is minimally affected by changes in utility prices. In contrast, the bio-PG process shows a moderate sensitivity: utility price changes impact its OPEX by less than 10%, while a 30% increase in glycerol prices leads to an approximate 23% rise in OPEX. For the bio-MTBE process, 95% of its OPEX is attributed to the bio-IBN process, which is highly sensitive to utility price fluctuations. Biomass price variations also influence the OPEX of bio-MTBE, though to a lesser extent. An important point is the shift in product and byproduct profiles and corresponding revenues (see Figures S1, S2 and Table S15). For instance, in the bio-PG process, ethylene glycol (MEG) and methanol are produced instead of dipropylene glycol (D-PG) and tripropylene glycol (T-PG). The bio-MTBE process generates additional byproducts such as CO_2_, n-butenes and furfural. As a result, compared to the fossil-based processes, the bio-PG process sees only a minor revenue decrease (c.a. 2 MEUR/y lower). In comparison, the bio-MTBE (total revenue of 284 MEUR/y) achieves double the revenue thanks to the added value of the new byproducts and utilities.

The MSPs of the fossil-based polyol and PG are close to their current market prices, while for MTBE, it is about 30% higher than the market price (see Table S5). Note that for the assessment at the process level, PO and TBA were assumed to be purchased externally. However, in the existing propylene cluster, the same company produces TBA and MTBE, and TBA is used internally for MTBE production (see Figure 1). This integration would reduce the MSP of MTBE, making it price-competitive (see fossil-based cluster Table 3 and Section 3.2). The MSPs of CO_2_-based polyol and bio-PG are 20–30% lower than fossil-based processes, while the MSP of bio-MTBE is 1.6 times higher, primarily due to the higher CAPEX and OPEX of the bio-IBN process (see Table 1).

The CO_2_-based polyol and bio-PG processes use less water (TWC) than their fossil-based counterparts (see Table S16). In contrast, the bio-MTBE process consumes 20 times more water, primarily due to the high energy and water demands of the bio-IBN process. Regarding CO_2_ emissions (see Figure 4), fossil-based processes have no CO_2_ emissions (Scope 1), while CO_2_-based polyol and bio-MTBE processes do have emissions, but they are of ACS origin. Most CO_2_ emissions for both fossil- and ACS-based, processes fall under Scope 2, originating from utility consumption. However, compared to their fossil counterparts, the CO_2_-based polyol and bio-PG emit 37% and 2% less CO_2_ emissions, respectively. Again, due to the high energy-intensive nature of the bio-IBN process, it emits 14 times more CO_2_ emissions (Scope 2). Note that in this assessment, additional utilities were assumed to be supplied from current market conditions (i.e., fossil-based feedstocks) (see Section 2.3). If a defossilized utility supply is considered (see Table S6), Scope 2 emissions of CO_2_-based polyol and bio-PG processes would be significantly lower, c.a 90%. Because of the energy needs, Scope 2 emissions of the bio-MTBE process would remain higher (417 kt CO_2_-eq/y) than those of the fossil-based process, but only by a factor of two. This point highlights the need to defossilize utilities along with industrial processes.

**Figure 4:**
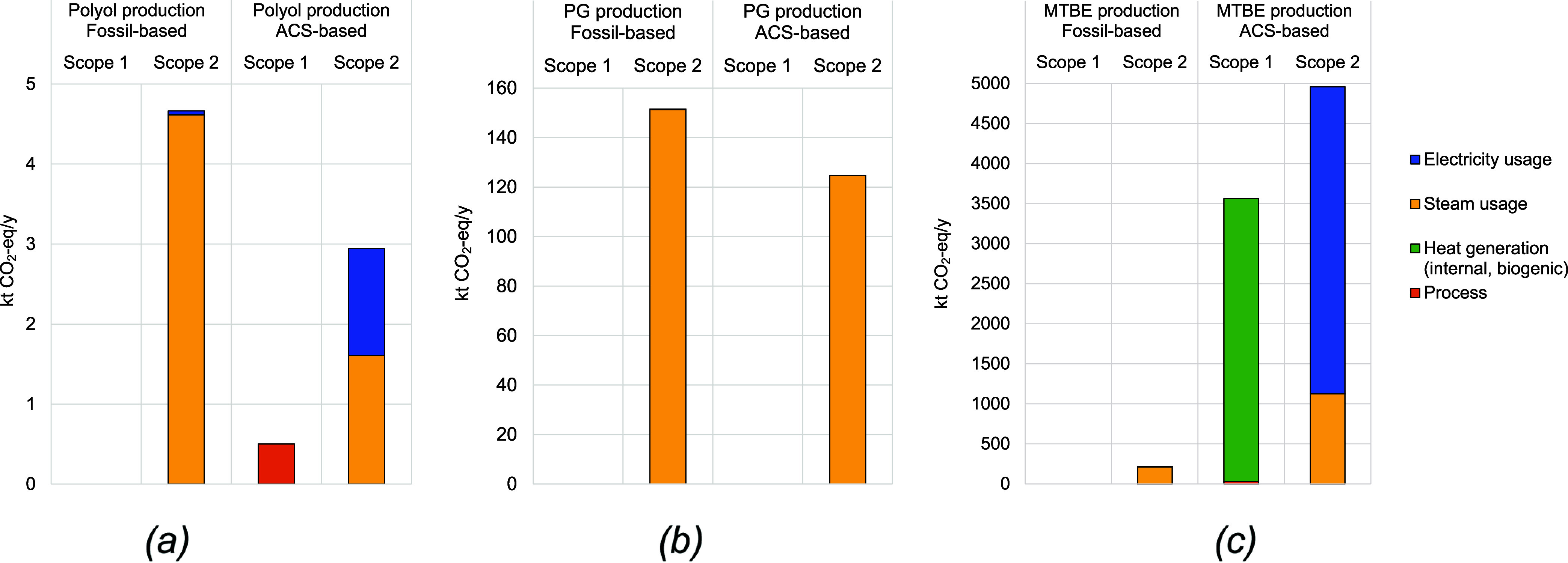
Total CO_2_ emissions of fossil- and ACS-based production processes of (a) polyol, (b) PG and (c) MTBE (for further details, refer to the Table S16).

In all cases, the bare land requirement (TL) of ACS-based processes is higher than that of fossil-based counterparts (see Table S16). The TL of the CO_2_-based polyol and bio-PG processes are 2 and 1.1 times higher, while the bio-MTBE process is c.a. 40 times higher. Thus, deploying ACS processes within the current cluster’s infrastructure would enlarge its bare land requirements. This point highlights the importance of assessing land constraints when integrating a new process into “crowded” existing clusters.

### 3.2 Cluster Level

#### 3.2.1 Case Study 1 – Defossilization of One DD at a Time

The results above show that the ACS-based processes require less to no fossil-based upstream chemicals compared to the reference case (see Section 3.1). When these processes are deployed individually in the propylene cluster, the structure and production of upstream fossil-based processes remain unchanged (see Table 2 and Figures S3, S5 and S7). This is because, in each case, either PO or TBA is still required to meet the existing demand for other DD in the cluster (see Section 3.1 and Figures S4, S6 and S8). However, under the assumption that no external market would exist for chemicals derived from fossil-based sources (Section 2.4.2), unused PO and/or TBA in the cluster are treated as “waste”.

**Table 2:** Summary of the Structural Changes in Processes on the Cluster Level after the Individual Deployment of ACS Processes – Case Study 1 (Based on Figures S3, S5 and S7)

	Deployed process		
Fossil-based propylene cluster	CO_2_-based polyol	Bio-PG	Bio-MTBE
Olefin production	unchanged	unchanged	unchanged
C4 Isomerization	unchanged	unchanged	unchanged
PO/TBA production	unchanged	unchanged	unchanged
Isobutene production	unchanged	unchanged	**removed**
Waste-fired boiler	unchanged	unchanged	unchanged
MTBE production	unchanged	unchanged	unchanged
PGME production	unchanged	unchanged	unchanged
PG production	unchanged	**removed**	unchanged
Polyol production	**removed**	unchanged	unchanged

The differences in the energy profiles between the fossil- and the alternative clusters are driven by the utility profiles of ACS processes (see Section 3.1). The bio-PG cluster shows (see Table 3) the lowest utility consumption among all one-to-one cases. This is due to the lower utility needs of the bio-PG process and its production of 300 TJ/y of LPS, which can cover c.a. 5% of the LPS demand of upstream fossil-based processes. The bio-MTBE process produces excess steam, fully covering the cluster’s needs for LLPS and HPS (see Table S11).

**Table 3:**
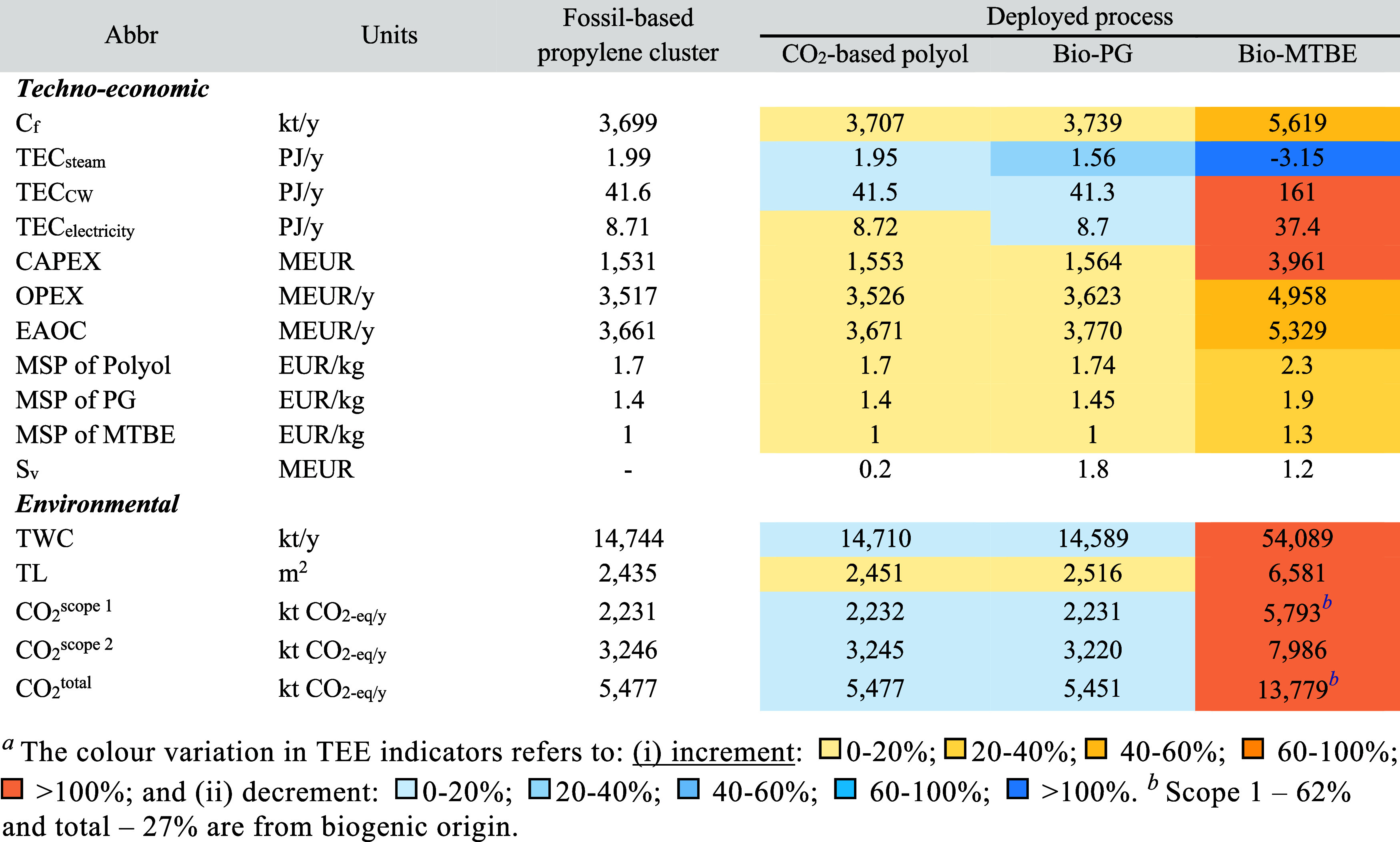
Techno-Economic and Environmental Indicators for the Assessment of the Propylene Cluster before and after the Individual Deployment of ACS Processes – Case Study 1 (Based on the Indicators from Table S7)

Although the OPEX of the CO_2_-based polyol and bio-PG processes are lower at the process level (see Section 3.1), their cluster level OPEX are higher due to (i) waste treatment costs for unused fossil-based PO (i.e., 5 MEUR for polyol, 13 MEUR for PG); (ii) the ACS processes require additional chemicals that are not produced in the cluster (i.e., CO_2_, glycerol). In the bio-MTBE cluster, 30% of the total OPEX of the cluster comes from the bio-MTBE process itself (mainly due to bio-IBN), while only 2% comes from the waste treatment of unused TBA (504 kt/y). The bio-PG cluster generates 2 MEUR/y less revenue than the fossil-based cluster. In contrast, the bio-MTBE cluster generates 352 MEUR/y more revenue, largely due to the additional byproducts (see Table S15).

After integrating the CO_2_-based polyol and bio-PG processes, the MSPs of the chemicals produced in the cluster remain unchanged compared to the fossil-based case. This is because the total CAPEX and OPEX of the cluster increase by less than 3% in both cases. In contrast, defossilizing the MTBE process results in about 30% higher MSPs, primarily due to the high costs associated with bio-IBN. The salvage value (S_v_) of the equipment of the replaced fossil-based processes is notably small, covering only 0.5% of the CAPEX for the new ACS-based clusters.

Scope 1 CO_2_ emissions of alternative clusters remain unchanged in the cluster with CO_2_-based polyol and bio-PG (Table 3). However, in the case of the cluster with the bio-MTBE process, Scope 1 emissions are 2.6 times higher than in the fossil-based cluster; 60% of these emissions are biogenic, originating from the bio-IBN on-site heat generation unit. Scope 2 CO_2_ emissions and TWC in the defossilized clusters are directly affected by the energy consumption and production profiles of the integrated ACS-based processes (see Section 3.1). Note that in all individual cases, Scope CO_2_ emissions could be lower by 85% if the heat and electricity supplied are defossilized along with the deployment of ACS-based processes (see Table S6).

#### 3.2.2 Case Study 2 – Defossilization of Several DD at the Same Time

The results show that simultaneously deploying all three ACS processes significantly impacts upstream production processes (see Figure 5). Starting with PO/TBA coproduction, 38% of the originally PO and 83% of the TBA production rates are no longer required for synthesizing the DD (see Section 3.1). However, due to the assumed limited operational flexibility of the chemical processes (see Section 2.4.2), PO production could only be reduced by 30%. Since PO and TBA are coproduced in the same process, this also constrains and limits the reduction in TBA.

**Figure 5:**
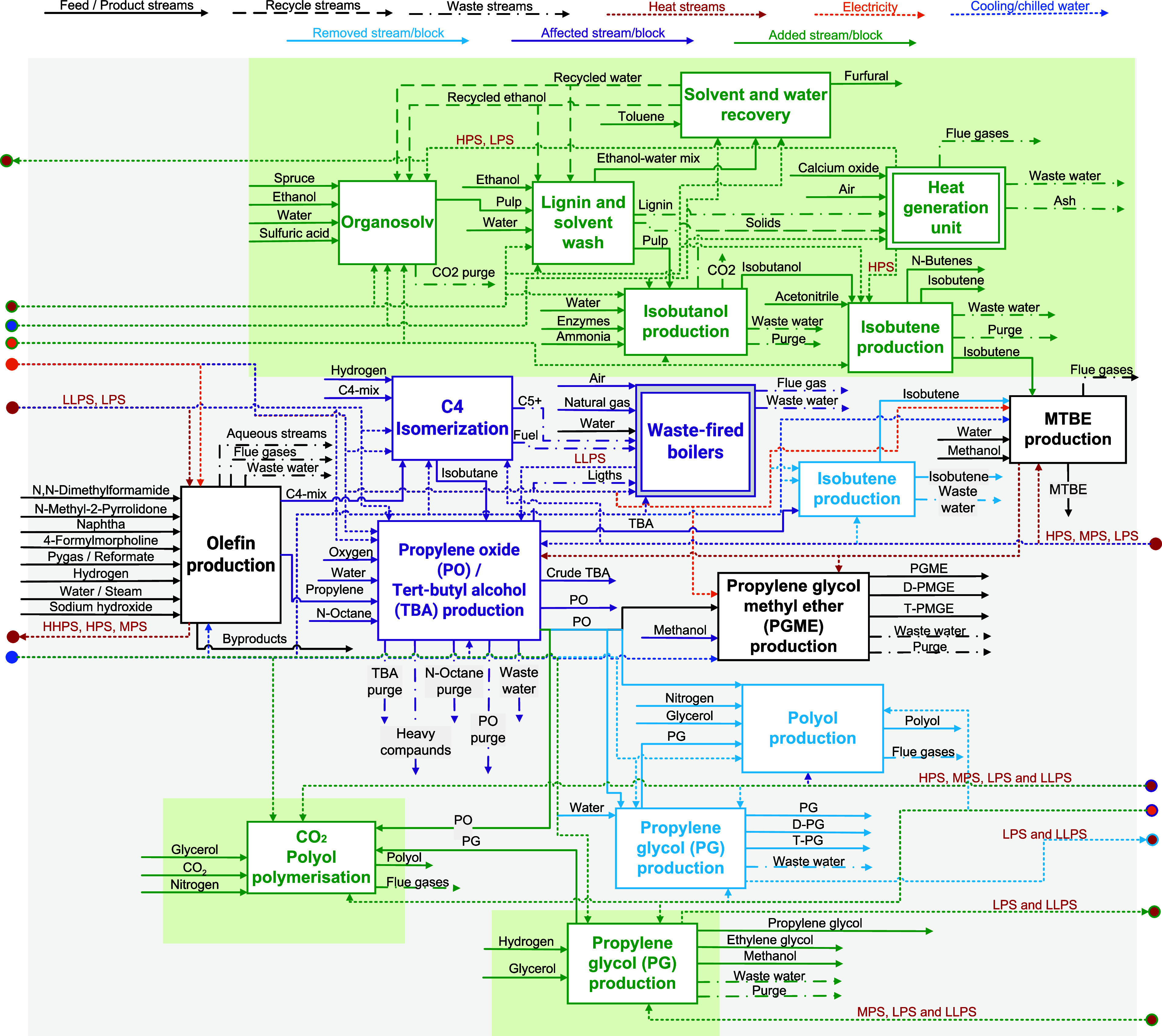
Map of changes inside the cluster after simultaneously integrating CO_2_-based polyol, bio-PG and bio-MTBE processes (i.e., isobutene production is changed to bio-IBN, MTBE production remains the same). The figure shows the processes/streams that would disappear (in blue), the ones that stay but are affected (in purple) and the new ones (in green).

The PO/TBA process relies on several upstream chemicals and utilities, i.e., propylene (187 kt/y) from olefin production, isobutane (549 kt/y) from C4 isomerization and internal heat generated by waste-fired boilers (i.e., LLPS, 3.6 PJ/y). When PO production is reduced, the demand for isobutane and heat also decreases, thereby affecting the production capacities of the C4 isomerization process and the waste-fired boilers. The C4 isomerization process uses 93 kt/y of C4 mix from the olefin production (unchanged), and 483 kt/y of C4 mix is bought from the market, which is now reduced by 30% due to lower demand. Although less propylene is required for PO/TBA production, the olefin production remains unchanged. This is because the unit must still supply the same amount of the C4 mix to the C4 isomerization process. As a result, 57 kt/y of unused fossil-based propylene and 323 kt/y of unused TBA are generated and treated as waste, incurring disposal costs (see Figure S9).

Within the propylene cluster, olefin production is the largest consumer of fossil-based carbon feedstock (87%), followed by the C4 isomerization process (11%), (see Table S12). The reduction in the C4 mix supplied from the market results in 120 kt/y less fossil carbon entering the cluster. However, the total carbon feedstock of the ACS-based cluster remains high (Table 4), with 2,118 kt/y coming from ACS-based sources.

**Table 4:**
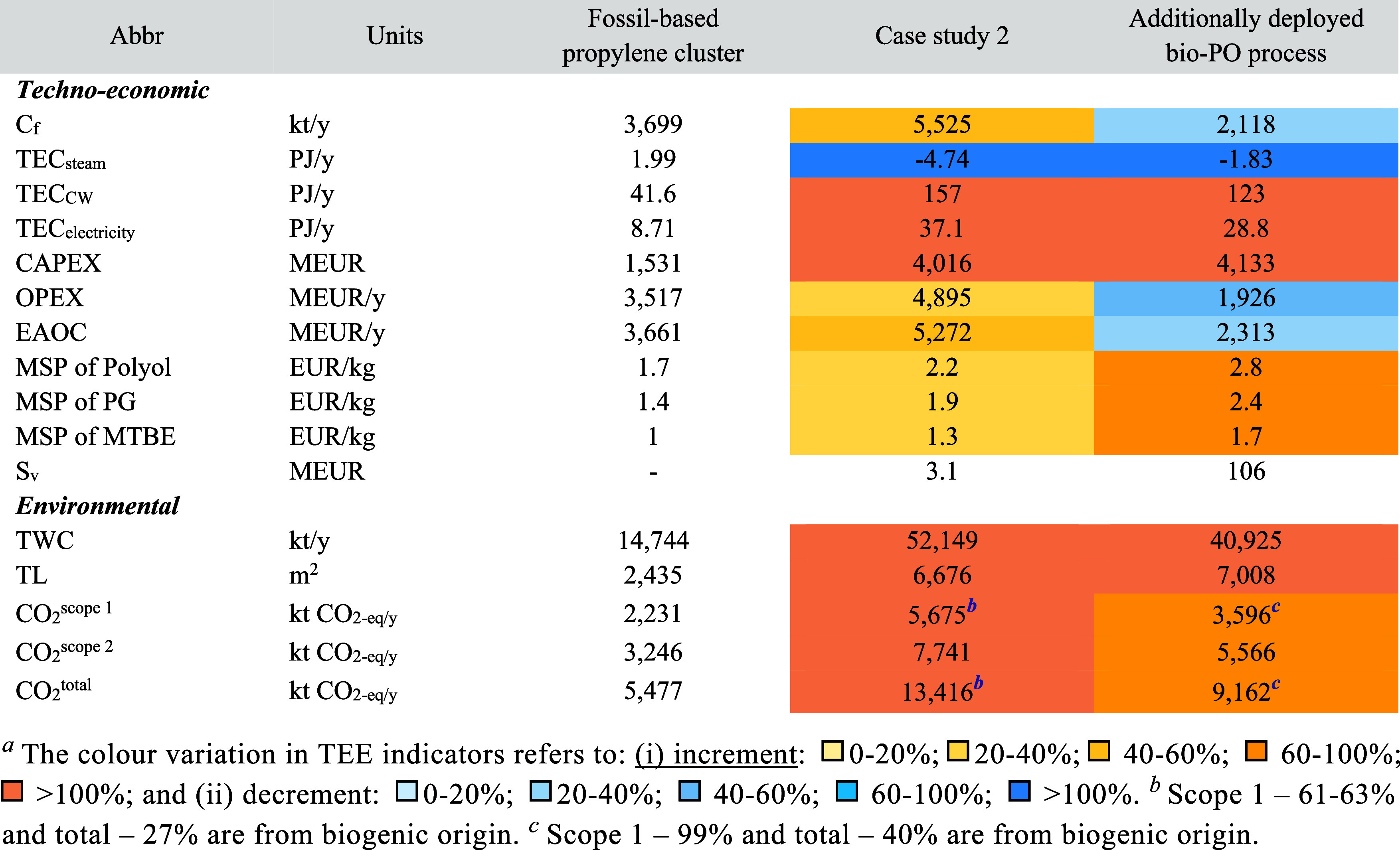
Techno-Economic and Environmental Indicators for the Assessment of the Propylene Cluster Before and After Simultaneous Deployment of the ACS Processes – Case Study 2 (Based on the Indicators from Table S7)

C4 isomerization and PO/TBA production correspond to c.a. 30% of the total energy demand of the cluster. Therefore, reducing these outputs, along with the removal of the fossil-based DD processes, leads to significant reductions in utility consumption: total steam by 20%, cooling water by 12%, and electricity by 4%. However, the new cluster requires c.a. 4 times higher cooling water and electricity consumption, mainly due to the bio-IBN process (see Table 4 and Figure 7b). Nonetheless, steam that was initially used by the removed fossil-based processes (see Table S10) can be repurposed to fully cover the LLPS and LPS needs of the ACS processes, and partially (26%) of the MPS needs. The bio-MTBE process can fully (LLPS and HPS) or partially (LPS, up to 24%) cover the needs of the upstream fossil-based processes. Nonetheless, additional MPS, cooling water and electricity are still required, mainly for the bio-MTBE process.

The CAPEX of the new cluster is 2.6 times higher than the fossil-based cluster, with 62% allocated to the bio-IBN process (see Table 4 and Figure 7c). In terms of OPEX, the majority (62%) is still driven by the olefin production process, while c.a. 30% is from the ACS-based processes. Notably, the OPEX reduction from the C4 isomerization, PO/TBA and waste-fired boilers amounts to 160 MEUR/y, while waste treatment costs for TBA and propylene disposal total 73 MEUR/y.

Although the ACS-based cluster generates a 10% higher revenue, the MSPs are still about 30% higher than those of the fossil-based case. This is caused by the higher CAPEX and OPEX, mainly due to the performance of the bio-MTBE process (see Section 3.1). The salvage value (S_v_) of the replaced fossil-based processes remains notoriously low (see Table 4).

Scope 1 CO_2_ emissions of the alternative cluster are c.a. 63% from a biogenic origin. Scope 2 emissions are 1.5 times higher than in the fossil-based cluster, mainly due to the bio-IBN process, which alone emits 2.4 times more CO_2_ emissions than the olefin production process (Figure 7e). Therefore, the total CO_2_ emissions (biogenic plus fossil) from the ACS-based cluster remain 2.4 times higher than those of the fossil-based cluster. Note that the Scope 2 CO_2_ emissions of Case study 2 can be up to 70% lower than those of the fossil-based cluster, if the utilities used were primarily delivered from a nonfossil origin (see Table S6). TWC is 4 times higher (Figure 7d), 76% consumed by the bio-IBN process and 15% by olefin production.

The ACS process for PGME production is excluded from the integration, as deploying an additional indirect route (see Figure 2) would likely expand the cluster further. However, the conversion of bio-PG to bio-PO, which can be internally reused for PGME and CO_2_-based polyol production, may lead to different outcomes. This option is explored in the next Section 3.2.3.

#### 3.2.3 Additional Deployment of Bio-PO Process

The deployment of the bio-PO production process, which uses bio-PG as feedstock, along with the other ACS-based processes described in Section 3.2.2, drastically changes the structure of the existing cluster. Specifically, seven out of nine fossil-based processes are replaced (see Table 4 and Figure 6). This transformation is primarily driven by the combined impact of deploying the bio-PO and bio-IBN processes, which eliminate the need for the fossil-based PO/TBA process, olefin production, C4 isomerization and waste-fired boilers. As a result, the propylene (187 kt/y) and isobutane (549 kt/y) previously used in PO/TBA production are no longer required. C4 mix demand is reduced by 483 kt/y (from the market) and 93kt/y from olefin production, used in the C4 Isomerization process. Due to the removal of the PO/TBA process, TBA (100 kt/y) is no longer supplied to the market. All products from olefin production are no longer produced in the propylene cluster (for more details, please refer to Table S4, olefins). The fossil-based MTBE and PGME processes remain unchanged. The newly deployed bio-PO production process needs to meet a PO demand of 153 kt/y, which is required by the CO_2_-based polyol and PGME processes and the market. To achieve this, the bio-PG production needs to be scaled up by a factor of 3.5 to produce 283 kt/y of bio-PG. This volume covers both the requirements for bio-PO production and the market demand for bio-PG (80kt/y) (see Figure S10).

**Figure 6:**
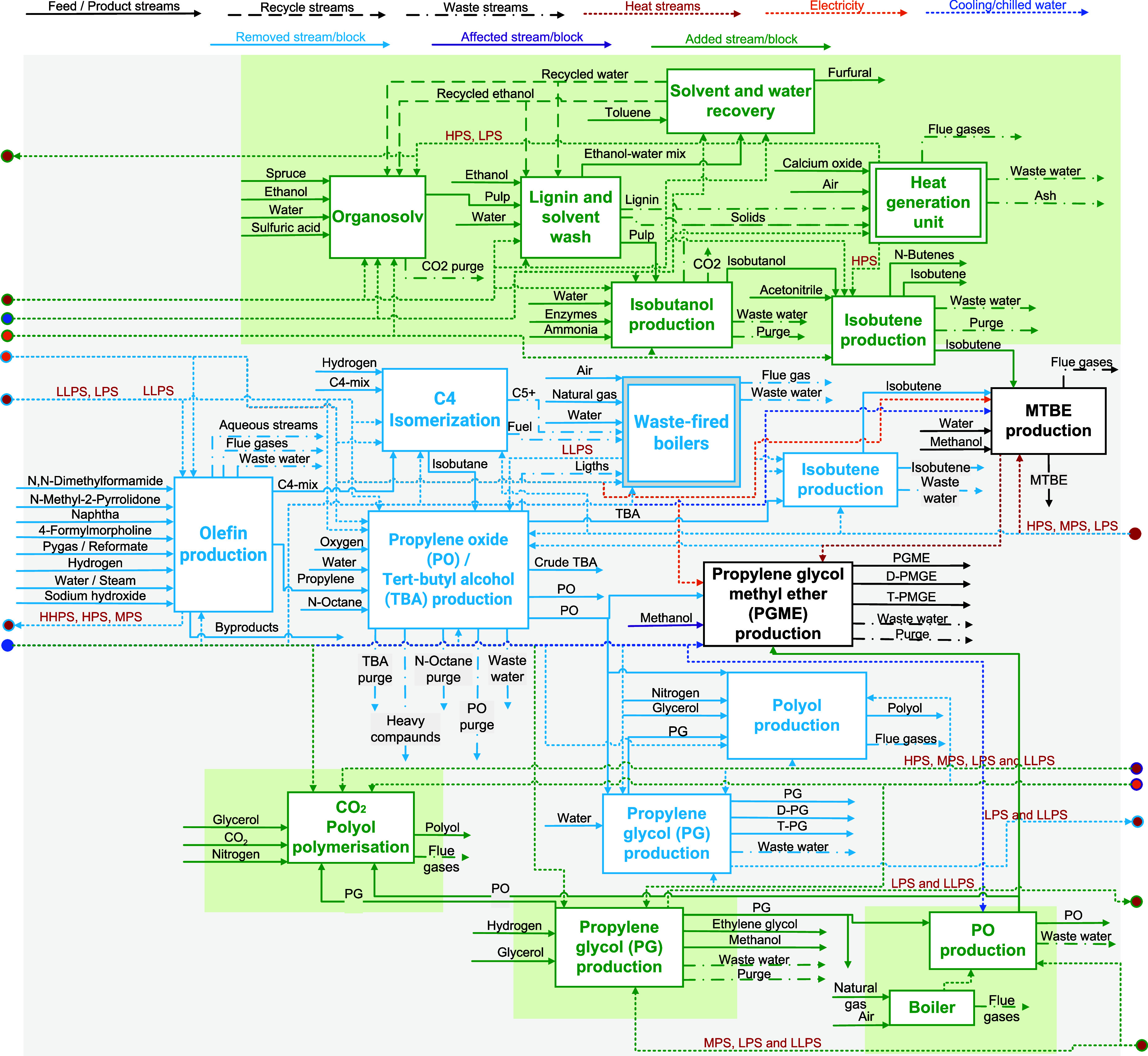
Map of changes inside the cluster after additionally integrating the bio-PO process. The figure shows the processes/streams that would disappear (in blue), the ones that stay but are affected (in purple) and the new ones (in green).

Compared to the fossil-based cluster (see Table 4 and Figure 7a), the ACS-based cluster now uses 43% less total carbon feedstock, all of which is 100% derived from ACS-based sources. The alternative cluster also consumes 20% less cooling water and electricity than the configuration in Section 3.2.2, due to the removal of olefin production, C4 Isomerization and PO/TBA production. However, it still needs 3 times more cooling water and electricity than the fossil-based cluster, mainly due to the bio-IBN process (see Figure 7b). Steam integration improves efficiency. LLPS, LPS, and MPS previously supplied to the removed processes can be reused in the deployed ACS-based processes. This reuse fully covers the needs for LPS, LLPS, and 34% of MPS, while excess steam can be sold to the market. The bio-PO process (see Figure 6) includes an on-site boiler fueled by natural gas, which supplies 57% of its heating demand, with the remaining supplied from the market.

**Figure 7:**
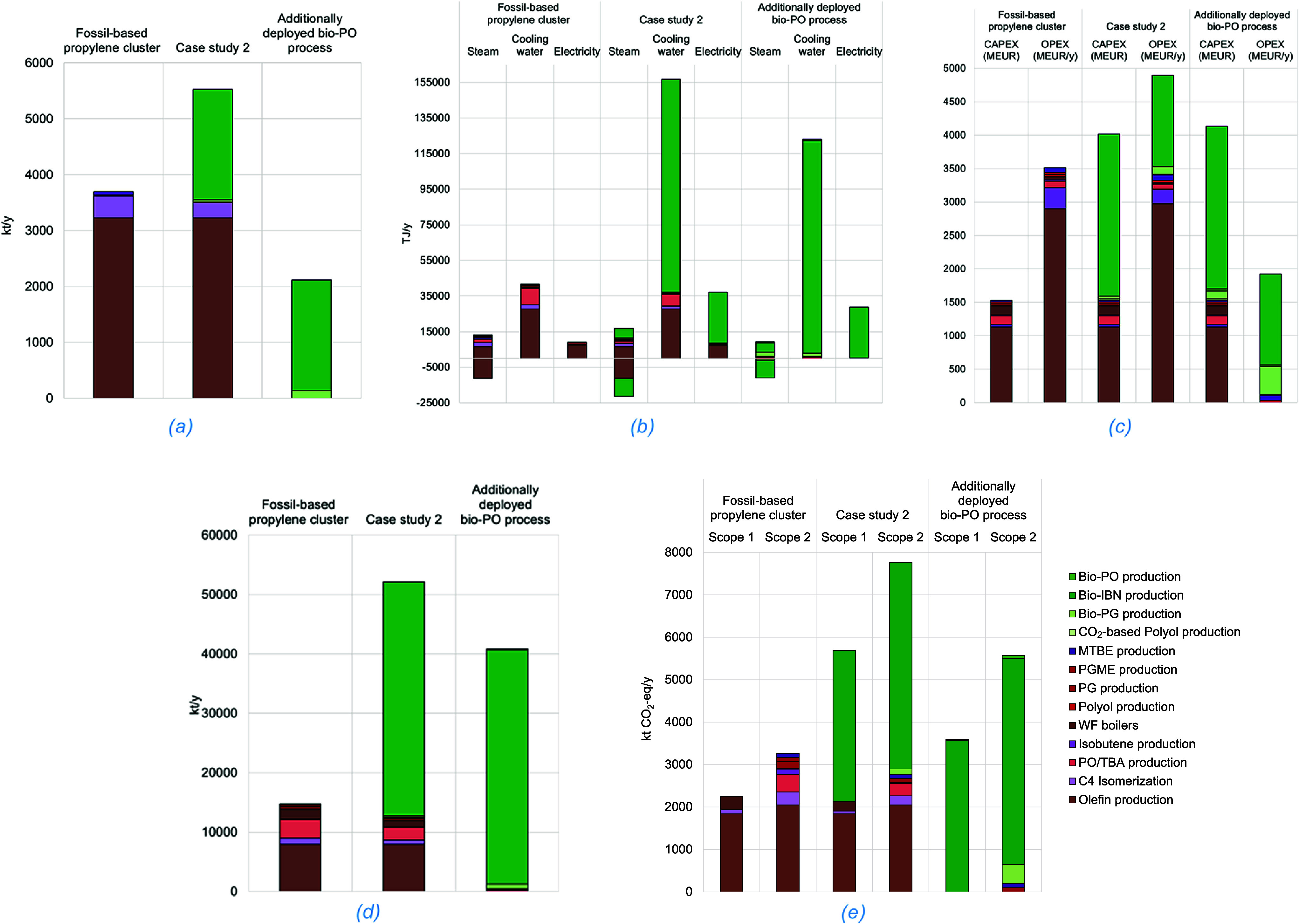
Techno-economic and environmental indicators for the assessment of the propylene cluster before and after deployment of the ACS processes simultaneously – Case study 2: (a) carbon feedstock; (b) power/energy requirements; (c) CAPEX and OPEX; (d) water consumption; (e) CO_2_ emissions.

Additional deployment of the bio-PO process hardly changes the CAPEX of the alternative cluster (see Table 4 and Figure 7c), which remains 2.7 times higher than that of the fossil-based cluster, with 63% of the CAPEX attributed to the ACS-based processes. Note that this assessment does not account for the costs of retrofitting or reusing the equipment, which could affect the ACS-based cluster’s economic performance. Due to the removed processes, the OPEX is c.a. 2.7 times lower than in Section 3.2.2, representing 53% of the OPEX of the removed fossil-based processes, 85% of which were due to olefin production.

The cluster’s revenue is now 2.6 times lower than in Case study 2 without bio-PO (see Section 3.2.2), primarily due to removing the olefin production process, which previously accounted for 70% of the total revenue in the fossil-based cluster. Moreover, in the current configuration, 100% of the revenue of the cluster is generated by ACS-based DD processes. As a result, to achieve a payback period of 25 years, all expenses must be distributed across these ACS processes, which produce a smaller and different portfolio of products and byproducts compared to the original fossil-based configuration or in the alternative cluster in Case study 2 (see Section 3.2.2). Therefore, the MSPs are higher, by almost 35% and 27%, respectively (Table 4).

Less than 1% of Scope 1 CO_2_ emissions are from a fossil-based origin, due to the natural gas boiler on-site for bio-PO production (see Table 4). However, Scope 2 emissions are higher than those of the fossil-based cluster due to the CO_2_ emissions from the bio-IBN process (Figure 7e). Employing defossilized utility sources could reduce the Scope 2 emissions to 452 kt CO_2_-eq/y, corresponding to 14% of the Scope 2 emissions from the fossil-based case. Compared to Case study 2 without the bio-PO process use (see Section 3.2.2), the TWC is 26% lower, but it is still 2.8 times higher (i.e., due to the bio-IBN process) than that of the fossil-based cluster.

The results from Case study 2 show that, while additional deployment of the bio-PO process along with the other three ACS-based DDS could drive the full defossilization of the cluster, it also introduces significant economic and environmental trade-offs. This highlights the importance of assessing the ACS technologies not as a stand-alone process, but also in the context of their integration into existing industrial clusters.

## 4 Conclusions

The study assesses the performance of an existing propylene cluster after integrating ACS-based processes to defosilize downstream derivatives (DD) production. Two case studies are used to explore different deployment strategies of ACS-based: (i) one DD production process at a time (i.e., individually), and (ii) multiple processes at the same time (i.e., simultaneously).

The results from Case study 1 (individual deployment) show that replacing fossil-based DD production processes, one at a time, with ACS-based alternatives does not significantly impact upstream production. However, upstream chemicals no longer required for ACS-based production, such as PO and/or TBA, are now produced in excess in the cluster. This raises a question about the fate of fossil-derived chemicals produced but not used within the cluster. Under an ambitious approach to foster defossilization, their production must be limited, or any surplus must be treated as waste with zero market value. In all cases, the ACS-based processes increase the cluster layout, affecting its performance, reflected in both CAPEX and OPEX increases. The individual deployment of relatively simple and energy-efficient processes, such as CO_2_-based polyol and bio-PG, reduces energy and water consumption, resulting in lower Scope 2 emissions of the cluster.

Case study 2 shows that deploying multiple ACS-based processes together could potentially defossilize the cluster if deploying several ACS-based processes (simultaneously) affects or eliminates the same (fossil-based) upstream unit(s), causing a so-called cascading effect. In Case study 2, Section 3.2.2, a 30% reduction in the (fossil-based) PO/TBA production results in 37% less fossil-based carbon entering the cluster. The additional deployment of the bio-PO process for PGME and CO_2_-based polyol production, Section 3.2.3, ultimately removes olefin production, C4 isomerization, and PO/TBA production, resulting in a 100% ACS-based cluster. However, the byproducts from those processes are also removed, increasing the MSPs of the products in the ACS-based cluster (1.6 times higher). The new cluster results in three times higher capital investment than the fossil-based one. In both examples of Case study 2, while Scope 1 fossil-based CO_2_ emissions are lower than for the reference case, Scope 2 emissions are higher than the baseline because the model uses the current electricity and heat grid in the PoR. Suppose the utilities are also defossilized, the ACS-based cluster results in significantly lower CO_2_ emissions. This stresses the need to defossilize the utility supply along with downstream production.

Finally, several aspects require further investigation, including the availability of land in the existing clusters and the supply and logistics of ACS-based feedstocks. Additionally, the potential to reuse the equipment from removed fossil-based processes for ACS-based production and the environmental and economic implications of continuing to produce fossil-based chemicals no longer integrated in the cluster could be explored.

## Supplementary Material



## Notes

Literature review of ACS-based routes, Process data sheets (incl. Input data for TEE assessment (incl. allocation factors), Results from the modeling, Case study 1 (Structural changes and Mass balances), Case study 2 (Mass balances) (PDF)

## Abbreviations

ACS: Alternative carbon sources
CAPEX: Capital expenditures
DD: Downstream derivative
EAOC: Equivalent annual operating costs
HPS: High-pressure steam
IBN: Isobutene
LLPS: Very low-pressure steam
LPS: Low-pressure steam
MPS: Medium-pressure steam
MSP: Minimum selling price
MTBE: Methyl tert-butyl ether
OPEX: Operational expenditures
PG: Propylene glycol
PGME: Propylene glycol methyl ether
PJ: Petajoule
PO: Propylene oxide
PoR: Port of Rotterdam
TBA: Tert-butyl alcohol
TEE: Techno-economic and environmental analyses
TJ: Terajoule
TL: Total bare land requirement
TWC: Total water consumption
