# Supplementary material for: From Feedstock to Future Chemicals: Rethinking Carbon Sources in Industrial Propylene Clusters
Source: ACS Sustain Chem Eng. 2025 Oct 13;13(42):17869–80. doi: 10.1021/acssuschemeng.5c05287 (PMC12570266; doi:10.1021/acssuschemeng.5c05287)
Supplement: Supplementary file 1 [file sc5c05287_si_001.pdf]

Supporting Information (SI)

for

"From feedstock to future chemicals: Rethinking carbon sources in industrial propylene clusters"

Authors: Inna Stepchuk†,*, Mar Pérez-Fortes†, Andrea Ramírez‡

Affiliations:

† *Affiliation 1: Department of Engineering Systems and Services, Faculty of Technology, Policy and Management, Delft University of Technology, Jaffalaan 5, 2628 BX Delft, The Netherlands*

‡ *Affiliation 2: Department of Chemical Engineering, Faculty of Applied Sciences, Delft University of Technology, Van der Maasweg 9, 2628 CN Delft*

***Corresponding author:** Inna Stepchuk

email: i.stepchuk@tudelft.nl

Supplementary Information file consists of:

- 20 pages;
- 10 figures;
- 16 tables.

Table of Contents

Table of Figures S2
Table of Tables S2
Literature review of ACS-based routes S3
Input data for the TEE assessment S5
Calculation of the allocation factors S7
Results from modelling and TEE assessment S8
Case study 1: Structural changes and mass balances after defossilisation S12
Case study 2: Mass balances after defossilisation S15
References S16

Table of Figures

Figure S1. Mass flows of the fossil-based propylene cluster S8
Figure S2. Mass flows of ACS-based production processes: (a) CO_2-based polyol, (b) bio-PG and (c) bio-MTBE S8
Figure S3. Map of changes inside the cluster after integrating the CO_2-based polyol process S12
Figure S4. Mass flows inside the propylene cluster after integrating the CO_2-based polyol process S12
Figure S5. Map of changes inside the cluster after integrating the bio-PG process S13
Figure S6. Mass flows inside the propylene cluster after integrating the bio-PG process S13
Figure S7. Map of changes inside the cluster after integrating the bio-MTBE process S14
Figure S8. Mass flows inside the propylene cluster after integrating the bio-MTBE process S14
Figure S9. Mass flows inside the propylene cluster after simultaneously integrating CO_2-based polyol, bio-PG and bio-MTBE processes S15
Figure S10. Mass flows inside the cluster after additionally integrating the bio-PO process S15

Table of Tables

Table S1. List of ACS-based routes found in the literature to synthesise isobutene (IBN) as feedstock for MTBE production S3
Table S2. List of ACS-based routes found in the literature to synthesise polyol S3
Table S3. List of ACS-based routes found in the literature to synthesise propylene glycol (PG) and propylene oxide (PO) S4
Table S4. Detailed data sheets of the processes used in the assessment S4
Table S5. Summary of prices considered for the economic assessment (year 2018) S5
Table S6. CO_2 emission rates for utility sources. S5
Table S7. List of the techno-economic and environmental indicators used for the assessment S6
Table S8. Salvage value of the removed equipment. S6
Table S9. Allocation factors for the assessment at the process level S7
Table S10. Power and heat requirements and cooling needs for the production processes S9
Table S11. Power and heat excess for the production processes S9
Table S12. Carbon feedstock entering the processes S9
Table S13. CAPEX and OPEX of the processes S10
Table S14. Sensitivity analysis of the economic calculations for the ACS-based processes used in the assessment. S10
Table S15. Revenue of the processes S11
Table S16. Environmental indicators of the processes S11

Literature review of ACS-based routes

Table S1. List of ACS-based routes found in the literature to synthesise isobutene (IBN) as feedstock for MTBE production. Reproduced or adapted with permission from [1]. Copyright 2025 Elsevier Ltd.

ACS	Route	Type of product	Product	Ref.
Biomass	Fast pyrolysis	Chemical building block	Biooil	2,3
Biomass	Anaerobic digestion	Chemical building block	Biogas	2,3
Biomass	Gasification	Chemical building block	Syngas	2,4
Biooil	Chemical looping/reforming	Chemical building block	Syngas	5
Biooil	Gasification	Chemical building block	Syngas	3,4
Biomass	Plasma processing	Chemical building block	Syngas	6
Biogas	Dry / steam reforming	Chemical building block	Syngas	5
Biogas	Biogas upgrading	Chemical building block	Methane	4
Biogas	Catalytic methanation	Chemical building block	Methane	4
Methane	Partial oxidation	Chemical building block	Syngas	7
Methane	Dry / steam reforming	Chemical building block	Syngas	5
Biomass	Mevalonate pathway	Intermediate	Isobutene	8,9
Syngas	Catalytic methanation	Chemical building block	Methane	4
Syngas	Biomethanation	Chemical building block	Methane	7,10
Syngas	Fisher-Tropsch process	Chemical building block	Propylene	7
Syngas	Catalytic synthesis	Chemical building block	Methanol	4,11
Syngas	Catalytic isosynthesis	Intermediate	Isobutene	12
Syngas	Fermentation	Chemical building block	Ethanol	4
Methane	Plasma processing	Chemical building block	Methanol	13
Methane	Partial oxidation	Chemical building block	Methanol	14
Methane	Monooxygenase conversion	Chemical building block	Methanol	15
Methanol	Methanol-to-olefins	Chemical building block	Ethylene	16
Methanol	Methanol-to-olefins	Chemical building block	Propylene	16
Ethylene	Direct/indirect hydration	Chemical building block	Ethanol	17,18
Ethanol	Dehydration + dimerization + metathesis	Chemical building block	Propylene	19
Propylene	Oxidation	Intermediate	TBA	20
TBA	Dehydration	Intermediate	Isobutene	21
Ethanol	Dehydrogenation	Chemical building block	Acetaldehyde	12
Acetaldehyde	Oxidation	Chemical building block	Acetic acid	12,22
Acetic acid	Ketonisation	Intermediate	Acetone	12,22
Acetone	Aldol condensation/dehydration/acid site C-C cleavage	Intermediate	Isobutene	12
Ethanol	Guerbet-type co-condensation	Chemical building block	Isobutanol	23
Methanol	Guerbet-type co-condensation	Chemical building block	Isobutanol	23
Biomass	Enzymatic hydrolysis and fermentation	Chemical building block	Isobutanol	7
Isobutanol	Dehydration	Intermediate	Isobutene	24
CO_2	Sabatier process	Chemical building block	Methane	25
CO_2	Co-electrolysis	Chemical building block	Syngas	26
CO_2	Reverse water gas shift	Chemical building block	Syngas	4
Biogas	Catalytic synthesis	Chemical building block	Methanol	27
CO_2	Direct/indirect hydrogenation	Chemical building block	Methanol	28
CO_2	Direct/indirect hydrogenation	Chemical building block	Ethylene	4

Table S2. List of ACS-based routes found in the literature to synthesise polyol.

ACS	Route	Type of product	Product	Ref.
CO_2	Co-polymerisation	Downstream derivative	Polyol	29
Wastes	Solvolysis	Downstream derivative	Polyol	30
Biomass	Ring-opening polymerisation	Downstream derivative	Polyol	31
Biomass	Polycondensation	Downstream derivative	Polyol	31
Biomass	Dehydration	Downstream derivative	Polyol	31

Table S3. List of ACS-based routes found in the literature to synthesise propylene glycol (PG) and propylene oxide (PO). Note that only biomass-based routes were identified.

ACS	Route	Type of product	Product	Ref.
Sorbitol	Catalytic hydrogenolysis	Downstream derivative	PGE	32
Glycerol	Catalytic hydrogenolysis	Downstream derivative	PGE	32
Biomass	Transesterification	Chemical building block	Glycerol	33
Biomass	Hydrolysis	Chemical building block	Glycerol	33
Lactic acid	Reduction	Downstream derivative	PGE	32
Glycerol	Catalytic distillation with hydrogen	Downstream derivative	PGE	34
PGE	Cyclodehydration	Intermediate	PO	32,35
PGE	Catalytic synthesis	Downstream derivative	Acetone	35
PGE	Catalytic epoxidation	Intermediate	PO	36,37
Biomass	Pyrolysis/cracking	Chemical building block	Propylene	38
Bioethanol	Dehydration/ demirization and metathesis	Chemical building block	Propylene	38
Syngas	Synthesis	Intermediate	PO	39
Isobutanol	Dehydration/cracking	Chemical building block	Propylene	40
Ethanol	Dehydration/cracking	Chemical building block	Propylene	40

Table S4. Detailed data sheets of the processes used in the assessment. Data sheets are uploaded to the Zenodo repository in a form of the excel sheets, containing the following information: the process flow diagrams, data used for the modelling including description of the processes and Aspen process flowsheets, assumptions, input data (including references) and output data (i.e., mass and energy balances, CAPEX, OPEX, land footprint).

ID	Process	Capacity, kt/y	Detailed process sheets
**In-house model (Fossil-based)**			
O1	Olefins	3,000 (Naphtha)	41
P1.1	C4 Isomerisation	549	42
P1	Propylene Oxide /Tert-butyl Alcohol (PO/TBA)	250 (PO) / 603 (TBA)	
M6.1	Isobutene	358	43
M6	Methyl tert-butyl ether (MTBE)	400	
U6	Waste-fired boiler (WFB)	1,671 (LLPS)	44
P4	Polyol production	70	45
P3.1	Propylene Glycol (PG)	80	46
P3	Propylene Glycol Methyl Ether (PGME)	90	47
**Deployed ACS-based DDs**			
CP4	CO_2-based polyol	70	48
BP3.1	Bio-Propylene Glycol	80	49
BP3.2	Bio-Propylene Oxide	60	50
BM6.1	Bio-Isobutene	358	51

Input data for the TEE assessment

Table S5. Summary of prices considered for the economic assessment (year 2018).

Name	Price	Unit	Ref.
Hydrogen	1,525	EUR/tonne	52
Oxygen	0.12	EUR/tonne	53
Natural gas	358	EUR/tonne	54
Water	0.27	EUR/tonne	55
Propylene	1,016	EUR/tonne	56
Octane	426	EUR/tonne	57
N-Butane	471	EUR/tonne	58
Methanol	358	EUR/tonne	59
Isobutene	1,608	EUR/tonne	60
Polyol	1,750	EUR/tonne	59
MTBE	1,029	EUR/tonne	61
Ethylene glycol	1,326	EUR/tonne	62
Propylene glycol	1,458	EUR/tonne	63
D-Propylene glycol	1,458	EUR/tonne	63
T-Propylene glycol	1,458	EUR/tonne	63
Propylene glycol methyl ether	1,458	EUR/tonne	63
D-Propylene glycol methyl ether	1,458	EUR/tonne	63
T-Propylene glycol methyl ether	1,458	EUR/tonne	63
TBA	1,217	EUR/tonne	64
Propylene oxide	1,633	EUR/tonne	65
Hazardous waste stream	192	EUR/tonne	66
Wastewater	1.5	EUR/tonne	67
Ash	50	EUR/tonne	66
Acetonitrile	1,585	EUR/tonne	68
Biomass: spruce wood	37	EUR/tonne	69
Calcium Oxide (quicklime)	288	EUR/tonne	70
Enzymes (Cellulase enzyme cocktail)	2,000	EUR/tonne	71
Sulfuric acid	47	EUR/tonne	72
Ammonia	317	EUR/tonne	73
Ethanol	1,926	EUR/tonne	74
Toluene	625	EUR/tonne	75
Nitrogen	0.21	EUR/tonne	76
Glycerol	855	EUR/tonne	77
Catalyst Zinc (Polyol)	3,058	EUR/tonne	78
Carbon dioxide	75-80	EUR/tonne	79
N-butene	1,013	EUR/tonne	80
Furfural	1,421	EUR/tonne	81
Very Low-Pressure Steam (LLPS)	47	EUR/tonne	82
Low-pressure steam (LPS)	47	EUR/tonne	82
Medium-pressure steam (MPS)	47	EUR/tonne	82
High-pressure steam (HPS)	47	EUR/tonne	82
Cooling water	0.0712	EUR/m^3	83
Electricity	0.0836	EUR/ kWh	84

Table S6. CO_2 emission rates for utility sources.

	Retrieved from the Aspen Plus models of utility processes*					Defossilised**
	**LLPS**	**LPS**	**MPS**	**HPS**	**Electricity**	**Steam/electricity**
kt CO_2/TJ	0.11	0.17	0.19	0.22	0.14	0.01

** taking into account natural gas-based steam boilers [85];*
*** considering the Swedish power sector, data from [86,87].*

Table S7. List of the techno-economic and environmental indicators used for the assessment. Applicable to process and cluster levels. Reproduced or adapted with permission from [1]. Copyright 2025 Elsevier Ltd.

Indicator	Formula	Data inputs
**Techno-economic**		
Carbon feedstock	$C_f = \sum_{i=1}^{n} m_i^{in} \cdot C_{wt\%_i}$	• m_i^{in} – mass of material entering the production process, kt/y;<br>• $C_{wt\%_i}$ – weight percent of the carbon in the chemical, wt%.
Net energy/power	$TEC = \sum_{i=1}^{n} E_i^{in} + \sum_{i=1}^{n} E_i^{out}$	• E_i^{in} – energy/power used in the production process, TJ/y;<br>• E_i^{out} – energy/power excess in the production process, TJ/y.
Capital expenditures	$CAPEX = ISBL + OSBL + ECC + +CC + WC$	• *ISBL* – inside battery limits (bare equipment costs), MEUR;<br>• *OSBL* – offsite battery limits, includes the costs of the additions (f.e. the site infrastructure); ~(0.3-0.4)**ISBL*, MEUR;<br>• *ECC* – engineering and constructions costs; ~(0.25-0.3)**ISBL*,MEUR;<br>• *CC* – contingency chargers ~0.1**ISBL*, MEUR;<br>• *WC* – working capital ~0.15**ISBL*, MEUR.
Operational expenditures	$OPEX = VCOP + DCOP + OCOP$	• *VCOP* – variable costs of production, MEUR/y;<br>• *DCOP* – direct costs of production, MEUR/y;<br>• *OCOP* – other costs of production, MEUR/y.
Equivalent annual operating costs	$EAOC = OPEX + \frac{CAPEX \cdot i \cdot (1+i)^{sl}}{(1+i)^{sl} - 1}$	• *sl* – total number of years of service life, assumed 25 years;<br>• *CAPEX* – capital expenditures, MEUR;<br>• *OPEX* – operational expenditures, MEUR/y;<br>• *i* – interest rate, assumed 8%.
Minimum selling price	$MSP = {Revenue}/{m_{prod}}$	• *Revenue* – the income generated from all sales of goods, MEUR/y.
**Environmental**		
Total water consumption	$TWC = \sum_{i=1}^{n} m_{w_i}^{in} + \sum_{i=1}^{n} k_{steam} \cdot m_i^{steam} + +\sum_{i=1}^{n} k_{CW} \cdot m_i^{CW} - \sum_{i=1}^{n} m_i^{WW}$	• m_{wi}^{in} – mass of process water entering the production process, kt/y;<br>• k_{steam}– coefficient for steam loss, assumed 25%;<br>• m_i^{steam} – mass steam used in the production process, kt/y;<br>• k_{CW} – coefficient for cooling water loss, assumed 2%;<br>• m_i^{CW} – mass steam used in the production process, kt/y;<br>• m_i^{WW} – mass of waste water exiting the production process, kt/y.
Total bare land requirement	$TL = \sum_{k=1}^{n} A_k$	• $k = (1 \ldots n)$ – number of equipment used in the production;<br>• A_k – bare equipment area, m^2.
Scope 1: Process-related CO_2 emissions	$CO_2^{scope1} = \sum_{j=1}^{n} CO_{2j}^{out}$	• $j = (1 \ldots n)$ – number of gaseous waste streams exiting production;<br>• $CO_{2\,j}^{out}$ –emissions associated with the waste stream, kt CO_2-eq/y.
Scope 2: Energy-related CO_2 emissions	$CO_2^{scope2} = \sum_{u=1}^{n} CO_{2u}^{out}$	• $u = (1 \ldots n)$ – number of utility flows entering the production;<br>• $CO_{2\,j}^{out}$ – emissions produced from utilities used, kt CO_2-eq/y.
Total CO_2 emissions	$CO_2^{total} = CO_2^{scope1} + CO_2^{scope2}$	• CO_2^{scope1} – emissions (Scope 1), kt CO_2-eq/y;<br>• CO_2^{scope2} – emissions (Scope 2), kt CO_2-eq/y.

Table S8. Salvage value of the removed equipment.

Indicator	Definition	Formula	Data inputs	Ref.
Salvage value	The fraction of the original CAPEX at the end of the plant life.	$S_V = CAPEX \cdot (1 - i)^{sl}$	• i – rate of the depreciation;<br>• *sl* – total number of years of service life, assumed 25 years;<br>• *CAPEX*– capital expenditures in the fossil-based value chain, MEUR.	88,89

Calculation of the allocation factors

The formula for allocation follows Equation (9) in [90] and reads as follows (Equation 1):

$$Af = \frac{\text{Revenue}_i^{product}}{\sum_{i=1}^{n}\text{Revenue}_i^{product}} = \frac{\text{Price}_i^{product}\cdot\text{mass flow}_i^{product}}{\sum_{i=1}^{n}\text{Price}_i^{product}\cdot\text{mass flow}_i^{product}} \quad (1)$$

Table S9. Allocation factors for the assessment at the process level. The allocation factor is assigned at the process level, incorporating all products, byproducts, and utilities as components of the total revenue. For details on processes, refer to the Table S4.

Production process	Feedstock	Product	Name	To unit	Allocation factor
Olefin production	fossil	main	Propylene	to PO/TBA	0.06
C4 Isomerisation	fossil	main	Isobutane	to PO/TBA	1
PO/TBA production	fossil	main	PO	to polyol	0.1
				to PG	0.1
				to PGME	0.1
				market	0.06
	fossil	byproduct	TBA	to isobutene	0.54
				market	0.11
Isobutene production	fossil	main	Isobutene	to MTBE	0.73
				market	0.27
Waste-fired boilers	fossil	utility	LLPS	to PO/TBA	1
MTBE production	fossil	main	MTBE	market	1
Polyol production	fossil	main	Polyol	market	1
PG production	fossil	main	PG	market	0.9
		byproduct	D-PG	market	0.08
		byproduct	T-PG	market	0.01
PGME production	fossil	main	PGME	market	0.85
		byproduct	T-PGME	market	0.11
		byproduct	D-PGME	market	0.04
Polyol production	ACS	main	Polyol	market	1
PG production	ACS	main	PG	market	0.96
		byproduct	Methanol	market	0.01
		byproduct	Ethylene glycol	market	0.04
Isobutene production	ACS	main	Isobutene	to MTBE	0.45
				market	0.17
		byproduct	CO_2	market	0.05
		byproduct	Furfural	market	0.07
		byproduct	Butenes	market	0.01
		utility	LLPS	market	0.18
		utility	LPS	market	0.04
		utility	HPS	market	0.02

Results from modelling and TEE assessment

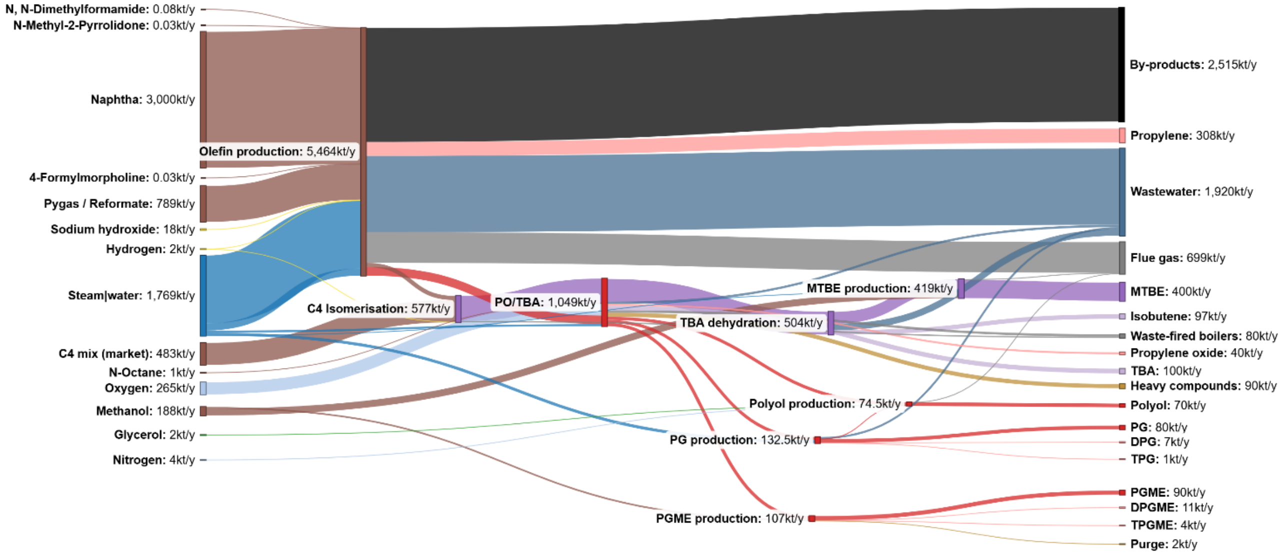

Figure S1. Mass flows of the fossil-based propylene cluster. For details on processes, refer to the Table S4.

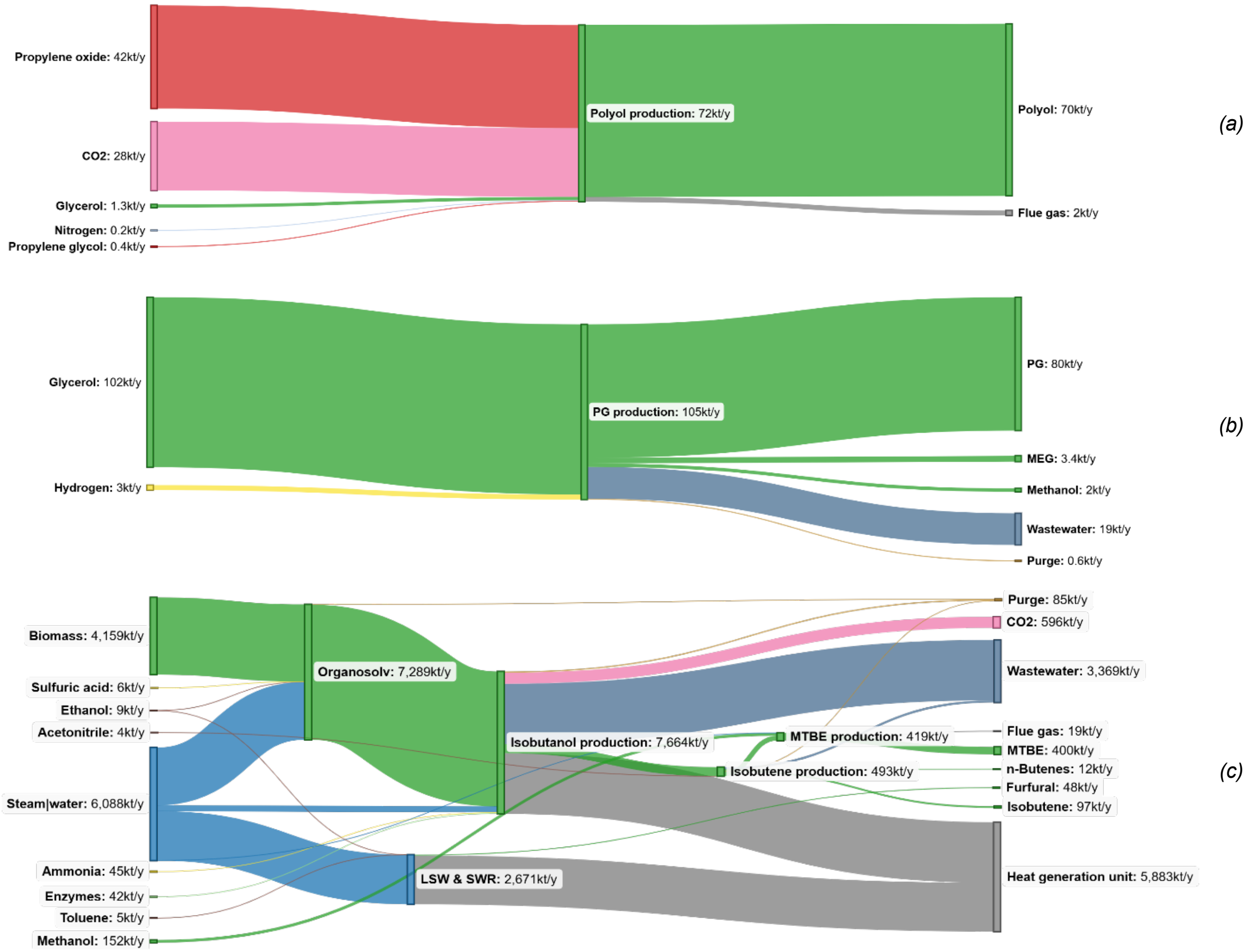

Figure S2. Mass flows of ACS-based production processes: (a) CO_2-based polyol, (b) bio-PG and (c) bio-MTBE. LSW & SWR - lignin and solvent wash unit and solvent and water recovery unit. For details on processes, refer to the Table S4.

Table S10. Power and heat requirements and cooling needs for the production processes. LLPS – very low-pressure steam; LPS – low-pressure steam; MPS – medium-pressure steam; HPS – high-pressure steam. For details on processes, refer to the Table S4.

Process	LLPS TJ/y	LPS TJ/y	MPS TJ/y	HPS TJ/y	Cooling water TJ/y	Electricity TJ/y
**Fossil-based**						
Olefin production	1,500	5,208	-	-	27,696	7,546
C4 Isomerisation	2,009	-	249	-	2,469	359
PO/TBA production	41	1,373	408	-	9,027	757
Isobutene production	-	-	618	-	500	40
WF boilers	-	-	-	-	-	-
Polyol production	36	5	-	-	158	-
PG production	-	-	799	7	800	2
PGME production	15	-	503	5	677	1
MTBE production	-	38	-	414	231	3
**ACS-based**						
CO_2-based Polyol production	-	9	-	-	64	10
Bio-PG production	17	-	659	-	493	-
Bio-IBN production	-	-	5,439	-	119,542	28,751
Bio-PO production	82	-	74	-	291	18

Table S11. Power and heat excess for the production processes. LLPS – very low-pressure steam; LPS – low-pressure steam; MPS – medium-pressure steam; HPS – high-pressure steam; HHPS – very high-pressure steam. For details on processes, refer to the Table S4.

Process	LLPS TJ/y	LPS TJ/y	MPS TJ/y	HPS TJ/y	HHPS TJ/y	Sent to
**Fossil-based**						
Olefin production	-	-	3,461	5,970	1,714	market
C4 Isomerisation	-	-	-	-	-	-
PO/TBA production	-	-	-	-	-	-
Isobutene production	-	-	-	-	-	-
WF boilers	3,568	-	-	-	-	PO/TBA production
Polyol production	-	-	-	-	-	-
PG production	90	7	-	-	-	market
PGME production	-	1	-	-	-	market
MTBE production	40	-	-	-	-	PGME production
	23	-	-	-	-	market
	249	-	-	-	-	PO/TBA production
**ACS-based**						
CO_2-based Polyol production	-	-	-	-	-	-
Bio-PG production	-	300	-	-	-	market
Bio-IBN production	7,759	1583	-	621	-	market
Bio-PO production	-	-	-	-	-	-

Table S12. Carbon feedstock entering the processes. The calculation was performed using indicators (see Table S7) employing the data from the processes (see Table S4).

Process	C_f kt/y	C_f kt/y
**Fossil-based**	stand-alone	part of the cluster
Olefin production	3,228	3,228
C4 Isomerisation	476	399
PO/TBA production	619	1
Isobutene production	318	0
WF boilers	91	42
Polyol production	43	1
PG production	42	0
PGME production	58	14
MTBE production	281	57
**ACS-based**		
CO_2-based Polyol production	34	8
Bio-PG production	40	40
Bio-IBN production	2,034	2,034
Bio-PO production	17	0

Table S13. CAPEX and OPEX of the processes. The calculation was performed using indicators (see Table S7) employing the data from the processes (see Table S4).

Process	CAPEX MEUR	OPEX MEUR/y	OPEX MEUR/y
**Fossil-based**		stand-alone	part of the cluster
Olefin production	1,130	2,900	2,900
C4 Isomerisation	37	361	309
PO/TBA production	129	1,040	107
Isobutene production	16	665	23
WF boilers	139	36	36
Polyol production	2	121	5
PG production	25	143	26
PGME production	26	156	34
MTBE production	26	520	78
**ACS-based**			
CO_2-based Polyol production	22	80	8
Bio-PG production	33	119	80
Bio-IBN production	2,430	1,366	1,366
Bio-PO production	13	132	10

Table S14. Sensitivity analysis of the economic calculations for the ACS-based processes used in the assessment.

	Unit	0%	10%	20%	30%
**CO_2-based Polyol production**					
Raw material costs, MEUR/y<br>Note: change in both glycerol and CO_2 prices	MEUR/y	72	72	72	73
OPEX	MEUR/y	80	80	80	81
Variation	%	0.0	0.4	0.8	1.2
Utility costs, MEUR/y<br>Note: change in both steam and electricity prices	MEUR/y	1	1	1	1
OPEX	MEUR/y	80	80	80	80
Variation	%	0.0	0.1	0.2	0.2
**Bio-PG production**					
Raw material costs, MEUR/y<br>Note: change in both glycerol and H_2 prices	MEUR/y	92	101	110	119
OPEX	MEUR/y	119	129	138	147
Variation	%	0	8	15	23
Utility costs, MEUR/y<br>Note: change in both steam and electricity prices	MEUR/y	17	19	21	22
OPEX	MEUR/y	119	122	125	128
Variation	%	0	2	5	7
**Bio-IBN production (stand-alone)**					
Raw material costs, MEUR/y<br>Note: change in biomass price	MEUR/y	281	296	312	327
OPEX	MEUR/y	1,366	1,549	1,564	1,579
Variation	%	0	13	15	16
Utility costs, MEUR/y<br>Note: change in both steam and electricity prices	MEUR/y	925	1,020	1,100	1,181
OPEX	MEUR/y	1,366	1,628	1,708	1,789
Variation	%	0	19	25	31
**MTBE production (incl. bio-IBN production)**					
Raw material costs, MEUR/y<br>Note: change in biomass price	MEUR/y	335	351	366	382
OPEX	MEUR/y	1,444	1,627	1,642	1,658
Variation	%	0	13	14	15
Utility costs, MEUR/y<br>Note: change in both steam and electricity prices	MEUR/y	939	1,034	1,116	1,198
OPEX	MEUR/y	1,444	1,708	1,789	1,871
Variation	%	0	18	24	30

Table S15. Revenue of the processes. Processes are considered to be a part of the cluster. The calculation was performed using data from the processes data sheets (see Table S4).

	**Products**	**Energy generated**
**Process**	**MEUR/y**	**MEUR/y**
**Fossil-based**		
Olefin production	2453	299
C4 Isomerisation	0	0
PO/TBA production	192	0
Isobutene production	156	0
WF boilers	0	0
Polyol production	123	0
PG production	128	2
PGME production	154	0
MTBE production	412	1
**ACS-based**		
CO_2-based Polyol production	123	0
Bio-PG production	122	7
Bio-IBN production	284	224
Bio-PO production	65	0

Table S16. Environmental indicators of the processes. The calculation was performed using indicators (see Table S7) employing the data from the processes (see Table S4).

	**CO_2 emissions**		**TWC**	**TL**
**Process**	**Scope 1**	**Scope 2**		
**Fossil-based**	kt/y	kt/y	kt/y	m^2
Olefin production	1,836	2,048	7,943	1,316
C4 Isomerisation	98	308	1,057	181
PO/TBA production	0	416	3,105	542
Isobutene production	0	122	97	46
WF boilers	297	0	1,671	112
Polyol production	0	4.6	55	8
PG production	0	153	383	75
PGME production	0	98	286	85
MTBE production	0	97	147	70
**ACS-based**				
CO_2-based Polyol production	0.5	3	21	15
Bio-PG production	0	127	228	81
Bio-IBN production	3,562	4,862	39,441	4,145
Bio-PO production	13	25	93	52

Case study 1: Structural changes and mass balances after defossilisation

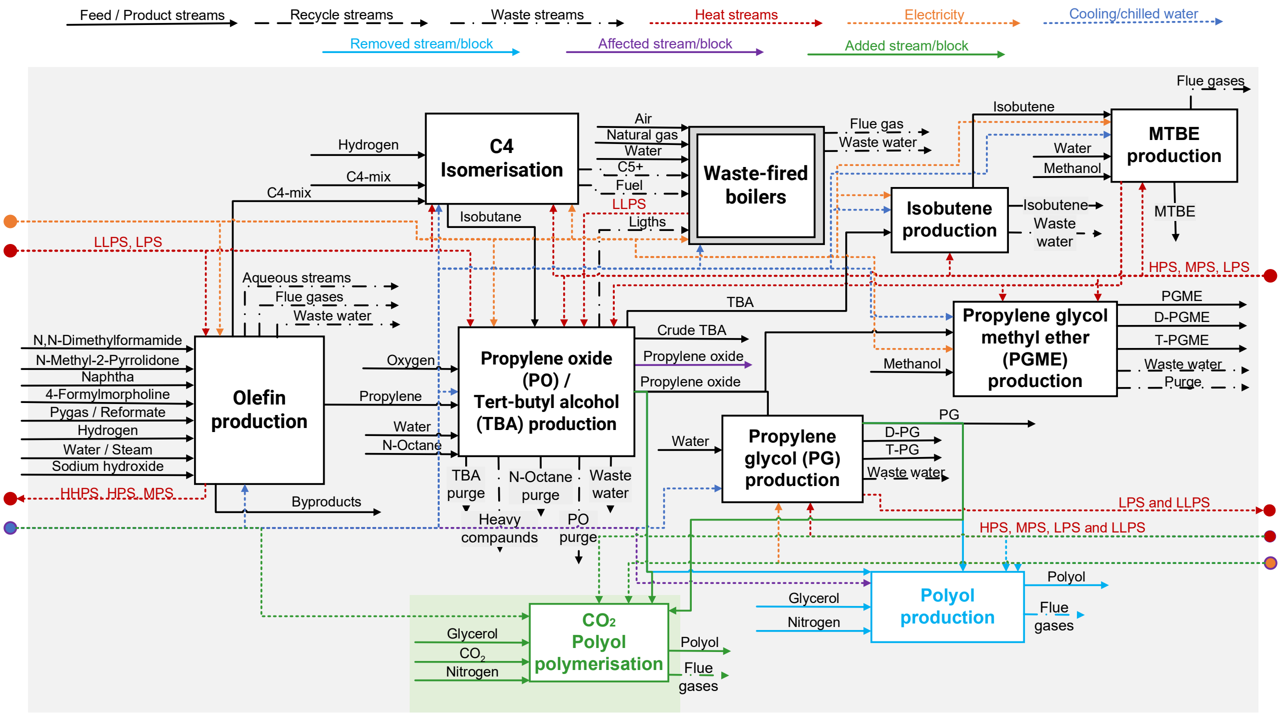

Figure S3. Map of changes inside the cluster after integrating the CO_2-based polyol process. The figure shows the processes/streams that would disappear (in blue), the ones that stay but are affected (in purple) and the new ones (in green). For details on processes, refer to the Table S4.

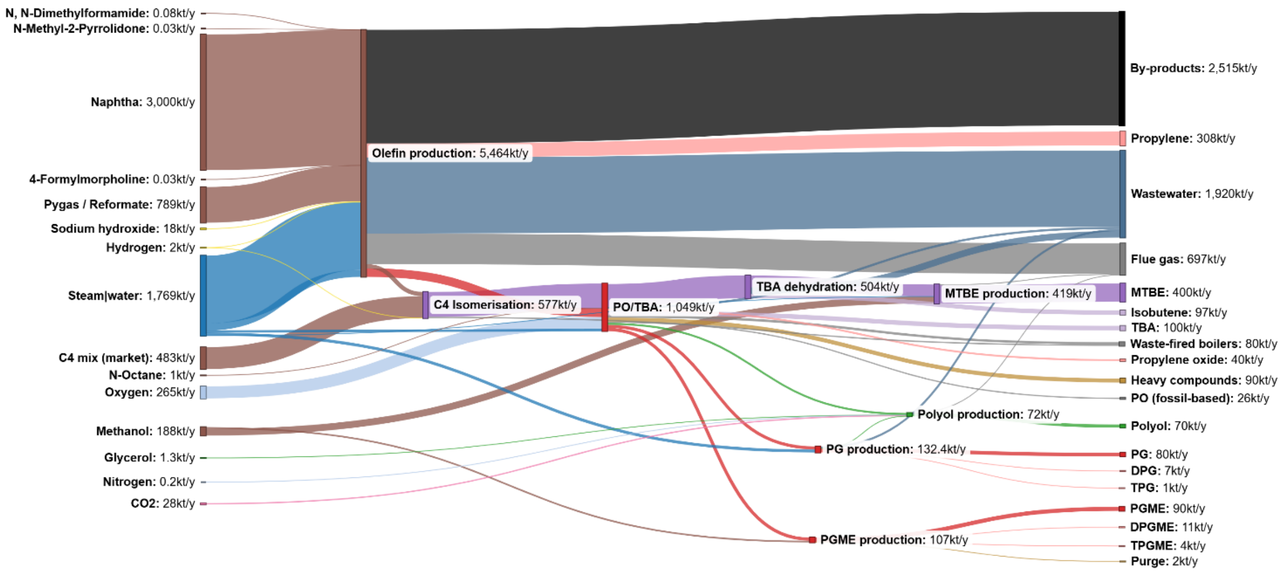

Figure S4. Mass flows inside the propylene cluster after integrating the CO_2-based polyol process. For details on processes, refer to the Table S4.

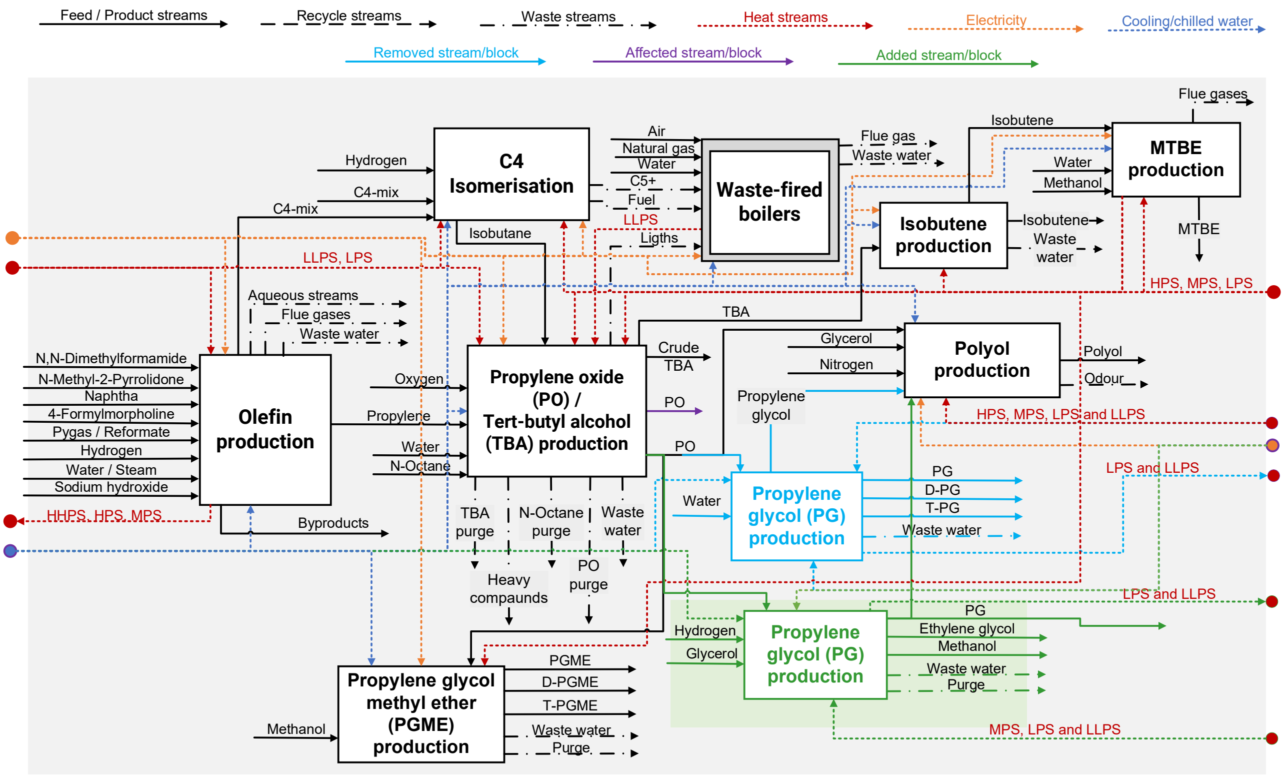

Figure S5. Map of changes inside the cluster after integrating the bio-PG process. The figure shows the processes/streams that would disappear (in blue), the ones that stay but are affected (in purple) and the new ones (in green). For details on processes, refer to the Table S4.

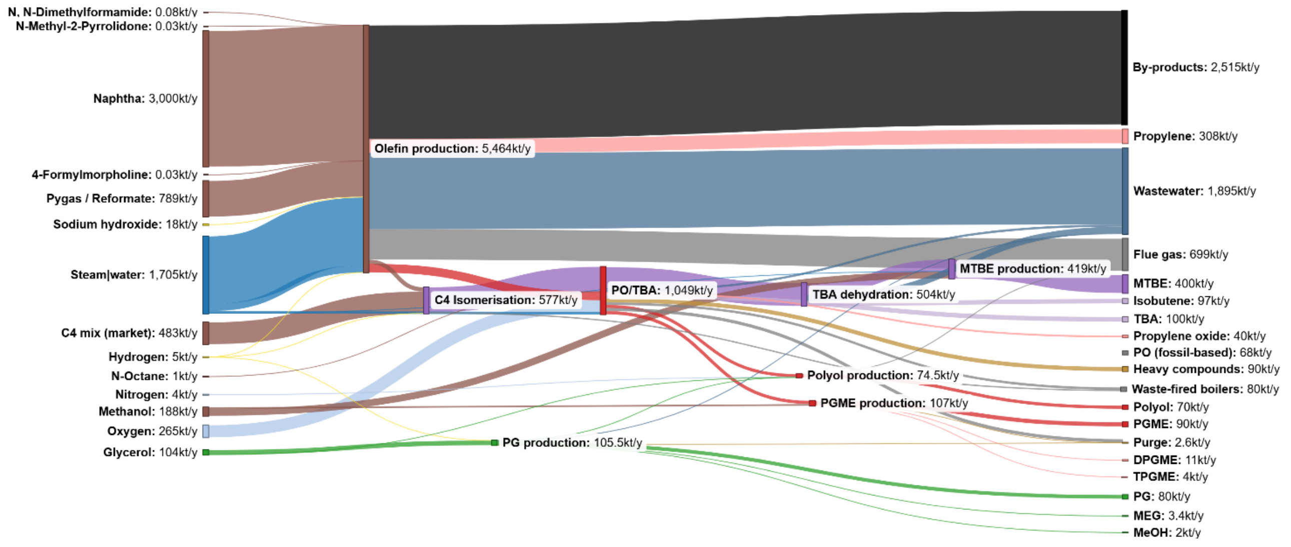

Figure S6. Mass flows inside the propylene cluster after integrating the bio-PG process. For details on processes, refer to the Table S4.

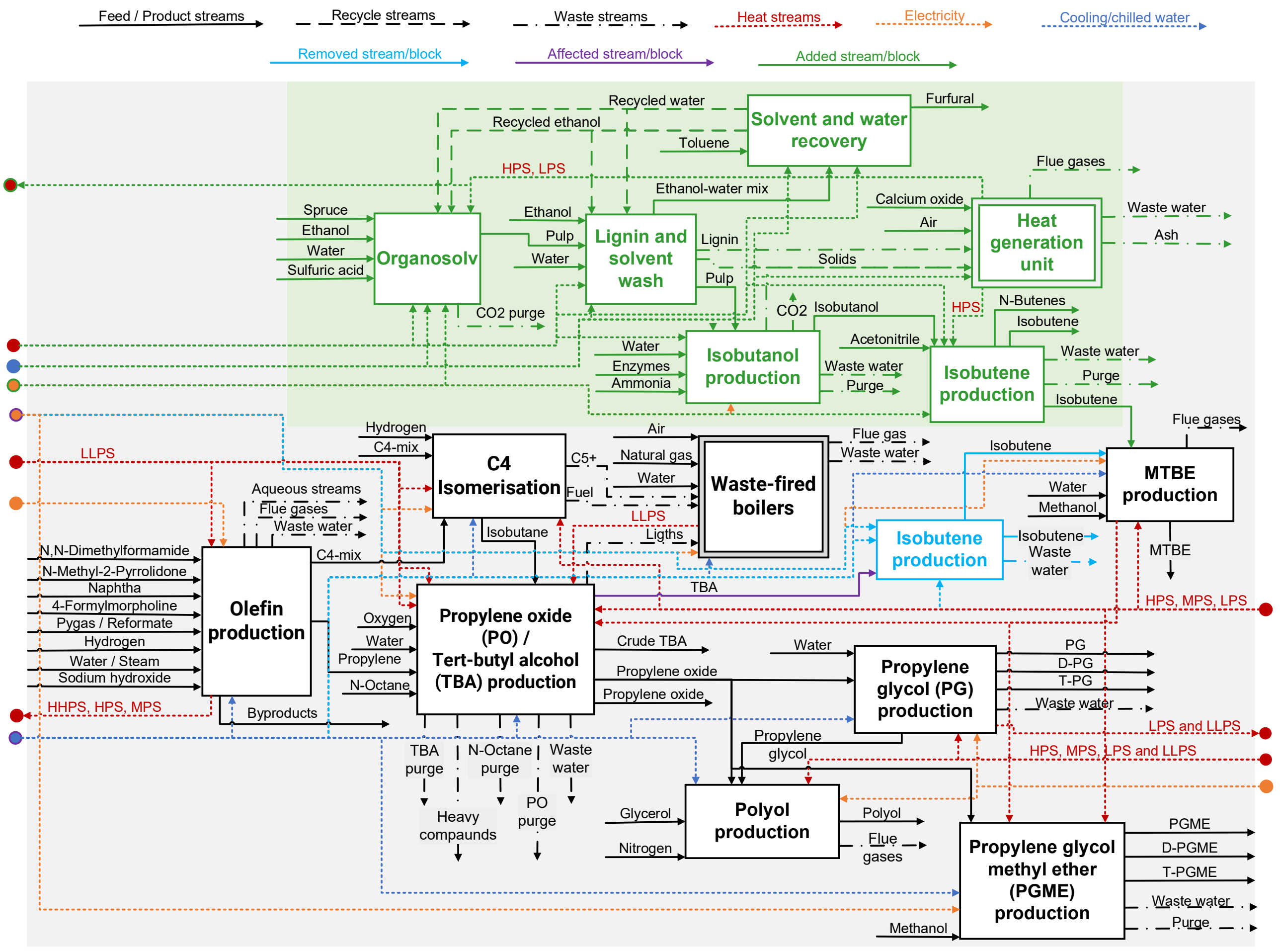

Figure S7. Map of changes inside the cluster after integrating the bio-MTBE process (i.e., isobutene production is changed to bio-IBN, MTBE production remains the same). The figure shows the processes/streams that would disappear (in blue), the ones that stay but are affected (in purple) and the new ones (in green). For details on processes, refer to the Table S4.

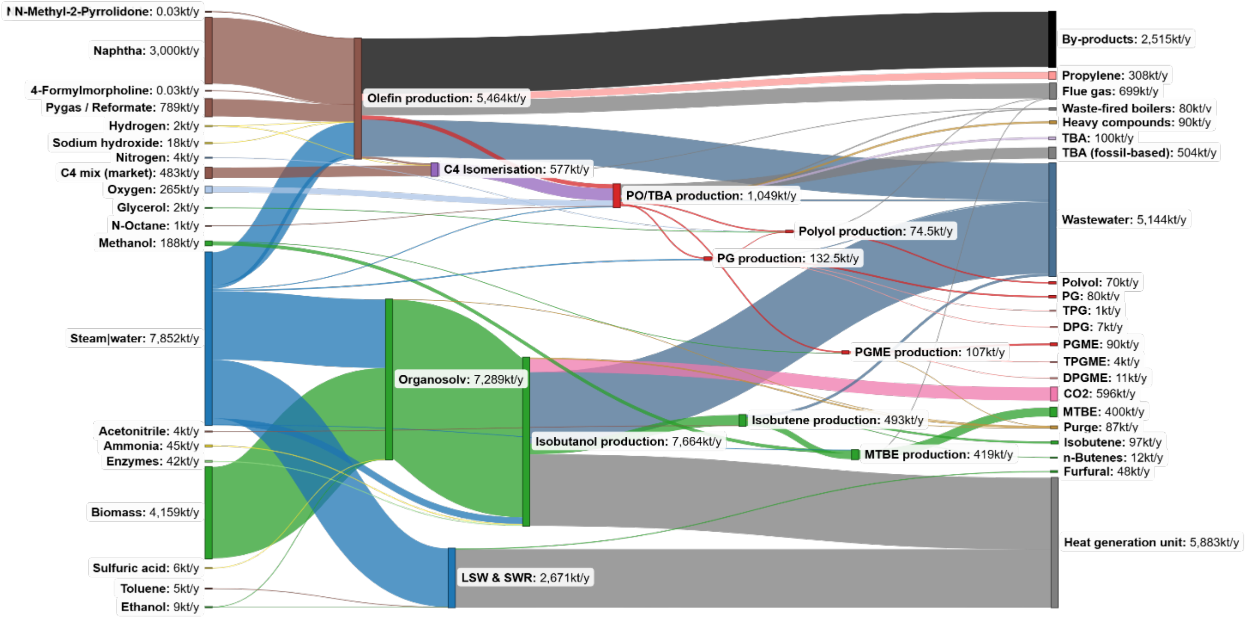

Figure S8. Mass flows inside the propylene cluster after integrating the bio-MTBE process. LSW & SWR - lignin and solvent wash unit and solvent and water recovery unit. For details on processes, refer to the Table S4.

Case study 2: Mass balances after defossilisation

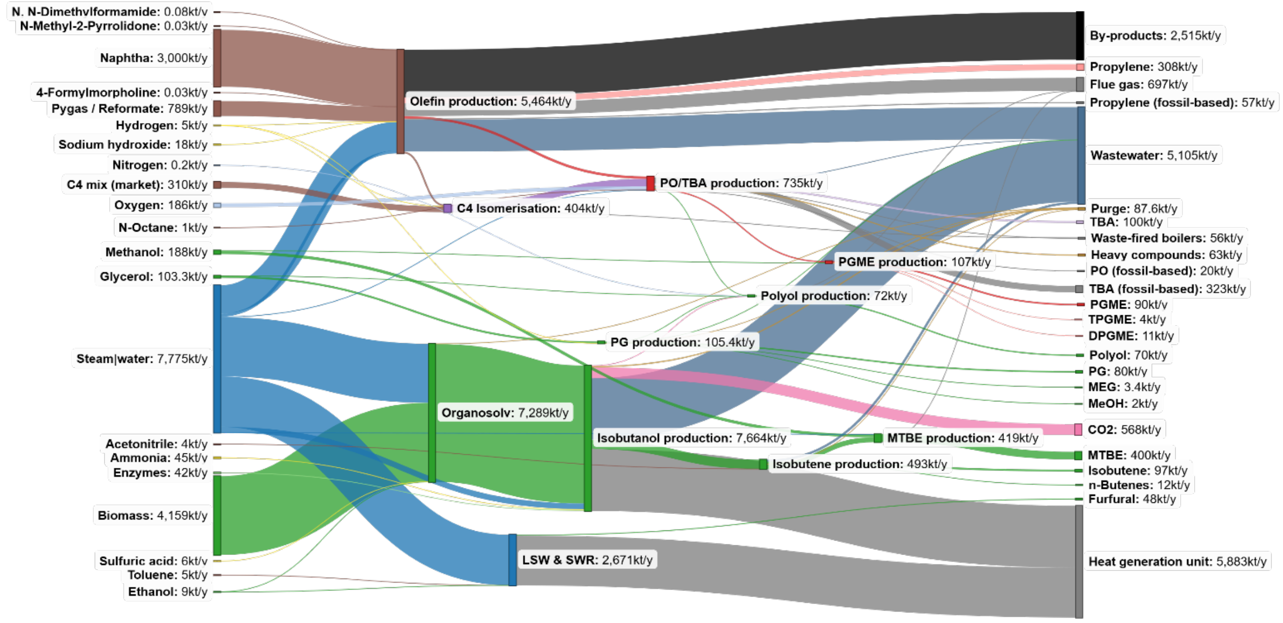

Figure S9. Mass flows inside the propylene cluster after simultaneously integrating CO_2-based polyol, bio-PG and bio-MTBE processes. LSW & SWR - lignin and solvent wash unit and solvent and water recovery unit. For details on processes, refer to the Table S4.

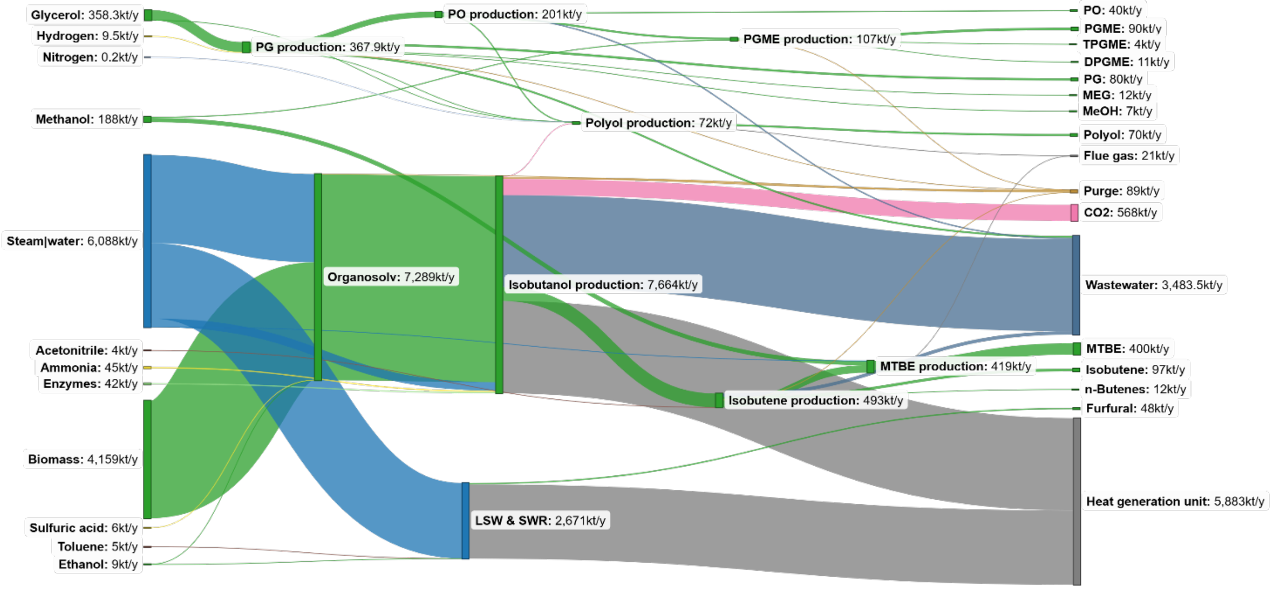

Figure S10. Mass flows inside the cluster after additionally integrating the bio-PO process. LSW & SWR - lignin and solvent wash unit and solvent and water recovery unit. For details on processes, refer to the Table S4.

References

(1) Stepchuk, I.; Pérez-Fortes, M.; Ramírez, A. Assessing Impacts of Deploying Bio-Based Isobutene for MTBE Production in an Existing Petrochemical Cluster. *J. Clean. Prod.* **2025**, *503*, 145114. https://doi.org/10.1016/j.jclepro.2025.145114.

(2) Thuijl, E. V.; Ree, R. V.; Lange, T. J. D. Biofuel Production Chains: Background Document for Modelling the EU Biofuel Market Using the BIOTRANS Model. **2003**, No. December, 1–40.

(3) Shamsul, N. S.; Kamarudin, S. K.; Rahman, N. A.; Kofli, N. T. An Overview on the Production of Bio-Methanol as Potential Renewable Energy. *Renew. Sustain. Energy Rev.* **2014**, *33*, 578–588. https://doi.org/10.1016/j.rser.2014.02.024.

(4) Schubert, T. Production Routes of Advanced Renewable <scp>C1</Scp> to <scp>C4</Scp> Alcohols as Biofuel Components – a Review. *Biofuels, Bioprod. Biorefining* **2020**, *14* (4), 845–878. https://doi.org/10.1002/bbb.2109.

(5) Ajdari, S. European Technology and Innovation Platform Bioenergy - Support of Renewable Fuels and Advanced Bioenergy Stakeholders 2. September 1, 2018, p 15. https://doi.org/10.3030/825179.

(6) Farsi, M. Biomass Conversion to Biomethanol. In *Advances in Bioenergy and Microfluidic Applications*; Elsevier, 2021; pp 231–252. https://doi.org/10.1016/B978-0-12-821601-9.00009-1.

(7) Gavrilescu, M. Biorefinery Systems. In *Bioenergy Research: Advances and Applications*; Elsevier, 2014; pp 219–241. https://doi.org/10.1016/B978-0-444-59561-4.00014-0.

(8) Marliere, P. Production of Alkenes by Enzymatic Decarboxylation of 3-Hydroxyalkanoic Acids. US20110165644A1, 2009. https://patents.google.com/patent/US20110165644A1/en.

(9) Saaret, A.; Villiers, B.; Stricher, F.; Anissimova, M.; Cadillon, M.; Spiess, R.; Hay, S.; Leys, D. Directed Evolution of Prenylated FMN-Dependent Fdc Supports Efficient in Vivo Isobutene Production. *Nat. Commun.* **2021**, *12* (1), 5300. https://doi.org/10.1038/s41467-021-25598-0.

(10) Menin, L.; Benedetti, V.; Patuzzi, F.; Baratieri, M. Techno-Economic Modeling of an Integrated Biomethane-Biomethanol Production Process via Biomass Gasification, Electrolysis, Biomethanation, and Catalytic Methanol Synthesis. *Biomass Convers. Biorefinery* **2023**, *13* (2), 977–998. https://doi.org/10.1007/s13399-020-01178-y.

(11) Iaquaniello, G.; Centi, G.; Salladini, A.; Palo, E. Waste as a Source of Carbon for Methanol Production. In *Methanol*; Elsevier, 2018; pp 95–111. https://doi.org/10.1016/B978-0-444-63903-5.00004-2.

(12) Berger, N. J.; Lindorfer, J.; Fazeni, K.; Pfeifer, C. The Techno-Economic Feasibility and Carbon Footprint of Recycling and Electrolysing CO2 Emissions into Ethanol and Syngas in an Isobutene Biorefinery. *Sustain. Prod. Consum.* **2022**, *32*, 619–637. https://doi.org/10.1016/j.spc.2022.05.014.

(13) Tang, Y.; Cui, Y.; Ren, G.; Ma, K.; Ma, X.; Dai, C.; Song, C. One-Step Synthesis of Methanol and Hydrogen from Methane and Water Using Non-Thermal Plasma and Cu-Mordenite Catalyst. *Fuel Process. Technol.* **2023**, *244*, 107722. https://doi.org/10.1016/j.fuproc.2023.107722.

(14) Park, M. B.; Park, E. D.; Ahn, W.-S. Recent Progress in Direct Conversion of Methane to Methanol Over Copper-Exchanged Zeolites. *Front. Chem.* **2019**, *7*. https://doi.org/10.3389/fchem.2019.00514.

(15) Khider, M. L. K.; Brautaset, T.; Irla, M. Methane Monooxygenases: Central Enzymes in Methanotrophy with Promising Biotechnological Applications. *World J. Microbiol. Biotechnol.* **2021**, *37* (4), 72. https://doi.org/10.1007/s11274-021-03038-x.

(16) Iaquaniello, G.; Centi, G.; Salladini, A.; Palo, E. Methanol Economy: Environment, Demand, and Marketing With a Focus on the Waste-to-Methanol Process. In *Methanol*; Elsevier, 2018; pp 595–612. https://doi.org/10.1016/B978-0-444-63903-5.00022-4.

(17) Britton, R. A. Direct Hydration of Ethylene to Ethanol. 3,686,334, 1972. https://patentimages.storage.googleapis.com/57/ab/7a/37d0017a3b3a72/US3686334.pdf.

(18) Carson, D. B. Process for the Manufacture of Ethanol from Ethylene. 4,296,261, 1980. https://patentimages.storage.googleapis.com/cd/de/04/c8ae0bb3612688/US4296261.pdf.

(19) Smith, J.; Sjodin, M. Simultaneous Dehydration, Dimerization, and Metathesis of C2-C5 Alcohols. WO2021067294A1, 2021. https://patentimages.storage.googleapis.com/90/4b/a7/d855422447dc56/WO2021067294A1.pdf.

(20) Baer, H.; Bergamo, M.; Forlin, A.; Pottenger, L. H.; Lindner, J. Propylene Oxide. In *Ullmann's Encyclopedia of Industrial Chemistry*; Wiley-VCH: Weinheim, Germany, 2012.

(21) Gupta, V. P. Liquid Phase Dehydration of Tertary Butylalcohol. 5,625,109, 1997.

(22) Kamm, B.; Kamm, M. Principles of Biorefineries. *Appl. Microbiol. Biotechnol.* **2004**, *64* (2), 137–145. https://doi.org/10.1007/s00253-003-1537-7.

(23) Zeng, F.; Hohn, K. L. Catalytic Conversion of Biomass-Derived Compounds to C4 Chemicals; 2019; pp 1–36. https://doi.org/10.1039/9781788016971-00001.

(24) Brouwer, G.; Loo, S. Van; Brouwer, B.; Scheppingen, Y. Van; Ransdorp, S.; Vries, S. F. De; Baets, P.; Noordende, H. V. 't; Kiel, J.; Ryan, C.; Drost, J.; Hennissen, B.; Versteeg, G.; Straathof, A.; Ramirez, A.; Lopez-Contrera, A.; Zirkzee, H. Iso-Butanol Platform Rotterdam (IBPR). **2016**.

(25) Stangeland, K.; Kalai, D.; Li, H.; Yu, Z. CO 2 Methanation: The Effect of Catalysts and Reaction Conditions. *Energy Procedia* **2017**, *105*, 2022–2027. https://doi.org/10.1016/j.egypro.2017.03.577.

(26) Lu, S.; Shi, Y.; Meng, N.; Lu, S.; Yu, Y.; Zhang, B. Electrosynthesis of Syngas via the Co-Reduction of CO2 and H2O. *Cell Reports Phys. Sci.* **2020**, *1* (11), 100237. https://doi.org/10.1016/j.xcrp.2020.100237.

(27) Grippi, D.; Clemente, R.; Bernal, M. P. Chemical and Bioenergetic Characterization of Biofuels from Plant Biomass: Perspectives for Southern Europe. *Appl. Sci.* **2020**, *10* (10), 3571. https://doi.org/10.3390/app10103571.

(28) Petrescu, L.; Galusnyak, S.-C.; Chisalita, D.-A.; Cormos, C.-C. Modelling and Simulation of Methanol Production and Conversion into Various Chemical Intermediates and Products; 2020; pp 553–558. https://doi.org/10.1016/B978-0-12-823377-1.50093-8.

(29) Fernández-Dacosta, C.; Van Der Spek, M.; Hung, C. R.; Oregionni, G. D.; Skagestad, R.; Parihar, P.; Gokak, D. T.; Strømman, A. H.; Ramirez, A. Prospective Techno-Economic and Environmental Assessment of Carbon Capture at a Refinery and CO2 Utilisation in Polyol Synthesis. *J. CO2 Util.* **2017**, *21*, 405–422. https://doi.org/10.1016/j.jcou.2017.08.005.

(30) Dürsen, F. G. C. Manufacturing of Polyols out of Alternative Sources – Chemistry and Engineering Aspects . https://www.keil-anlagenbau.de/fileadmin/user_upload/Manufacturing-of-polyols-out-of-alternative-sources_scientific-paper_Upload_20150423.pdf (accessed 2024-09-05).

(31) Sardon, H.; Mecerreyes, D.; Basterretxea, A.; Avérous, L.; Jehanno, C. From Lab to Market: Current Strategies for the Production of Biobased Polyols. *ACS Sustain. Chem. Eng.* **2021**, *9* (32), 10664–10677. https://doi.org/10.1021/acssuschemeng.1c02361.

(32) Haveren, J. van; Scott, E. L.; Sanders, J. Bulk Chemicals from Biomass. *Biofuels, Bioprod. Biorefining* **2008**, *2* (1), 41–57. https://doi.org/10.1002/bbb.43.

(33) Tan, H. W.; Abdul Aziz, A. R.; Aroua, M. K. Glycerol Production and Its Applications as a Raw Material: A Review. *Renew. Sustain. Energy Rev.* **2013**, *27*, 118–127. https://doi.org/10.1016/j.rser.2013.06.035.

(34) Clark, J.; Farmer, T.; Hunt, A.; Sherwood, J. Opportunities for Bio-Based Solvents Created as Petrochemical and Fuel Products Transition towards Renewable Resources. *Int. J. Mol. Sci.* **2015**, *16* (8), 17101–17159. https://doi.org/10.3390/ijms160817101.

(35) Yu, Z.; Xu, L.; Wei, Y.; Wang, Y.; He, Y.; Xia, Q.; Zhang, X.; Liu, Z. A New Route for the Synthesis of Propylene Oxide from Bio-Glycerol Derivated Propylene Glycol. *Chem. Commun.* **2009**, No. 26, 3934. https://doi.org/10.1039/b907530e.

(36) Liu, C.; Xin, J.; Tan, J.; Liu, T.; Kessler, M. R.; Zhang, J. Catalytic Conversion of Biomass-Derived 1,2-Propanediol to Propylene Oxide over Supported Solid-Base Catalysts. *ACS Omega* **2018**, *3* (8), 8718–8723. https://doi.org/10.1021/acsomega.8b01121.

(37) Santhanakrishnan, A.; Peereboom, L.; Miller, D. J. Catalytic Epoxidation of Propylene Glycol and Its Acetates. *Appl. Catal. A Gen.* **2018**, *561*, 19–27. https://doi.org/10.1016/j.apcata.2018.05.013.

(38) Phung, T. K.; Pham, T. L. M.; Vu, K. B.; Busca, G. (Bio)Propylene Production Processes: A Critical Review. *J. Environ. Chem. Eng.* **2021**, *9* (4), 105673. https://doi.org/10.1016/j.jece.2021.105673.

(39) Vora, B.; Pujado, P. Process for Producing Propylene Oxide. 5,599,955, February 22, 1996.

(40) Adam, C.; Minoux, Delphine Nesterenko, N.; Donk, S. Van; Dath, J.-P. Process to Make Propylene from Isobutanol by Dehydration and Subsequent Cracking. US20150239801A1, May 13, 2015.

(41) Manalal, J. T.; Tan, M.; Stepchuk, I.; Ibarra-González, P.; Pérez-Fortes, M.; Ramirez, A. R. Fossil-Based O1. Olefin Production (3000kt/y Naphtha) [Data Set]. Zenodo 2025. https://doi.org/10.5281/zenodo.14825234.

(42) Tan, M.; Stepchuk, I.; Manalal, J. T.; Ibarra-González, P.; Pérez-Fortes, M.; Ramirez, A. R. Fossil-Based P1. PO/TBA Production (PO:250/TBA:603 Kt) [Data Set]. Zenodo 2025. https://doi.org/10.5281/zenodo.14825844.

(43) Tan, M.; Stepchuk, I.; Manalal, J. T.; Ibarra-González, P.; Pérez-Fortes, M.; Ramirez, A. R. Fossil-Based M6. MTBE Production (400kt/y) [Data Set]. Zenodo 2025. https://doi.org/10.5281/zenodo.14825922.

(44) Tan, M.; Stepchuk, I.; Manalal, J. T.; Ibarra-González, P.; Pérez-Fortes, M.; Ramirez, A. R. Fossil-Based U6.I1&2 LLPs Production (1671 Kt) [Data Set]. Zenodo 2025. https://doi.org/10.5281/zenodo.14825977 (accessed 2025-02-06).

(45) Stepchuk, I.; Tan, M.; Manalal, J. T.; Ibarra-González, P.; Pérez-Fortes, M.; Ramirez, A. R. Fossil-Based P4. Polyol (70kt) Production [Data Set]. Zenodo 2025. https://doi.org/10.5281/zenodo.14906619.

(46) Stepchuk, I.; Manalal, J. T.; Tan, M.; Ibarra-González, P.; Pérez-Fortes, M.; Ramirez, A. R. Fossil-Based P31. PG (80kt) Production [Data Set]. Zenodo 2025. https://doi.org/10.5281/zenodo.14906587.

(47) Consonni, F.; Stepchuk, I.; Tan, M.; Manalal, J. T.; Ibarra-González, P.; Pérez-Fortes, M.; Ramirez, A. R. Fossil-Based P3. PGME (90kt) Production [Data Set]. Zenodo 2025. https://doi.org/10.5281/zenodo.14906547.

(48) Stepchuk, I.; Pérez-Fortes, M.; Ramirez, A. R. ACS-Based CP4. Polyol (70kt) Production [Data Set]. Zenodo 2025. https://doi.org/10.5281/zenodo.14910447.

(49) Stepchuk, I.; Pérez-Fortes, M.; Ramirez, A. R. ACS-Based BP31. PG (31kt) & (80kt) Production [Data Set]. Zenodo 2025. https://doi.org/10.5281/zenodo.14910522.

(50) Stepchuk, I.; Pérez-Fortes, M.; Ramirez, A. R. ACS-Based BP32. PO (23kt) & (60kt) Production [Data Set]. Zenodo 2025. https://doi.org/10.5281/zenodo.14910579.

(51) Stepchuk, I.; Pérez-Fortes, M.; Ramirez, A. R. ACS-Based BM61. Isobutene Production (360 Kt) [Data Set]. Zenodo 2025. https://doi.org/10.5281/zenodo.14826089 (accessed 2025-02-06).

(52) IEA. The Future of Hydrogen. *Futur. Hydrog.* **2019**, No. June. https://doi.org/10.1787/1e0514c4-en.

(53) Intratec. *Oxygen price | Current and forecast*. https://www.intratec.us/products/water-utility-costs/commodity/oxygen-price (accessed 2023-12-06).

(54) Statista. *Prices of natural gas for industry in the Netherlands from 2010 to 2021*. https://www.statista.com/statistics/595650/natural-gas-price-netherlands/ (accessed 2023-12-06).

(55) Intratec. *Process water cost | Current and forecast*. https://www.intratec.us/products/water-utility-costs/commodity/process-water-cost (accessed 2023-10-20).

(56) Intratec. *Propylene price | Current and forecast*. https://www.intratec.us/chemical-markets/propylene-price (accessed 2023-12-06).

(57) ChemicalBook. *n-octane*. https://www.chemicalbook.com/Price/N-OCTANE.htm (accessed 2023-12-06).

(58) Medium. *Butane price | United Kingdom — Q1 2023*. https://medium.com/intratec-products-blog/butane-price-united-kingdom-q1-2023-d210b724acba (accessed 2023-12-06).

(59) ICIS. *Europe Chemicals Outlook 2019*; 2019. https://s3-eu-west-1.amazonaws.com/cjp-rbi-icis/wp-content/uploads/sites/7/2019/01/24111814/EuropeChemicalOutlook_24012019-min.pdf (accessed 2022-01-19).

(60) Chemanalyst. *n-Butylene and isobutylene price: Trend and Forecast*. https://www.chemanalyst.com/Pricing-data/n-butylene-and-isobutylene-1149 (accessed 2023-10-20).

(61) Intratec. *MTBE price*. https://www.intratec.us/chemical-markets/mtbe-price?gclid=CjwKCAjw9J2iBhBPEiwAErwpefRtSQCCqtRk9rGoowETgYffDtIt9Gi-R0DEuUaZYokoxMP-do4kjBoCRyUQAvD_BwE (accessed 2023-04-25).

(62) Intratec. *Ethylene Glycol Prices | Current and Forecast*. https://www.intratec.us/chemical-markets/ethylene-glycol-price (accessed 2024-08-29).

(63) Intratec. *Propylene Glycol Prices | Historical and Current*. https://www.intratec.us/chemical-markets/propylene-glycol-price (accessed 2024-08-29).

(64) Intratec. *Tert butyl alcohol price | Current and historical*. https://www.intratec.us/chemical-markets/tert-butyl-alcohol-price (accessed 2023-10-20).

(65) Intratec. *Propylene oxide price | Current and forecast*. https://www.intratec.us/chemical-markets/propylene-oxide-price (accessed 2023-12-06).

(66) Turton, R.; Bailie, R. C.; Whiting, W. B.; Shaeiwitz, J. A. *Analysis, Synthesis, and Design of Chemical Processes*, Third Edit.; Pearson Education, Inc., 2009.

(67) Towler, G.; Sinnott, R. *Chemical Engineering Design: Principles, Practice, and Economics of Plant and Process Design*, 2nd ed.; Elsevier Ltd, 2013.

(68) Intratec. *Acetonitrile price | Current and forecast*. https://www.intratec.us/chemical-markets/acetonitrile-price (accessed 2023-10-20).

(69) GWMI. *Sweden: Sawlog and pulpwood prices see considerable drop in Q1/2020*. https://www.globalwoodmarketsinfo.com/sweden-sawlog-pulpwood-prices-see-considerable-drop-q1-2020/#:~:text=Pine logs cost now an,Sweden on a yearly basis (accessed 2023-12-06).

(70) ChemicalBook. *Calcium oxide | 1305-78-8*.

(71) Moncada, J.; Posada, J. A.; Ramírez, A. Comparative Early Stage Assessment of Multiproduct Biorefinery Systems: An Application to the Isobutanol Platform. *Bioresour. Technol.* **2017**, *241*, 44–53. https://doi.org/10.1016/j.biortech.2017.05.074.

(72) Intratec. *Sulfuric acid price | Current and historical*. https://www.intratec.us/chemical-markets/sulfuric-acid-price (accessed 2023-10-20).

(73) Intratec. *Ammonia price | Current and forecast*. https://www.intratec.us/chemical-markets/ammonia-price (accessed 2023-10-20).

(74) ICIS. *Chemical profile: Europe ethanol*. https://www.icis.com/subscriber/icb/chemicalprofile?commodityId=10170&regionId=10345#_=_.

(75) Intratec. *Toluene price | Current and forecast*. https://www.intratec.us/chemical-markets/toluene-price (accessed 2023-10-20).

(76) Intratec. *Nitrogen Price | Industrial Utilities*. https://www.intratec.us/products/water-utility-costs/commodity/nitrogen-price (accessed 2024-08-29).

(77) Intratec. *Glycerol Prices | Historical and Current*. https://www.intratec.us/chemical-markets/glycerol-price (accessed 2024-08-29).

(78) Intratec. *Zinc Prices | Current and Forecast*. https://www.intratec.us/chemical-markets/zinc-price (accessed 2024-08-29).

(79) Mikunda, T.; Neele, F.; Wilschut, F.; Hanegraaf, M. A Secure and Affordable CO2 Supply for the Dutch Greenhouse Sector. *Tno* **2015**, 38.

(80) Intratec. *Butene price | Current and forecast*. https://www.intratec.us/chemical-markets/butene-price (accessed 2023-10-20).

(81) Dalinyebo. *Furfural market prices*. https://dalinyebo.com/furfural-market-prices-2014/ (accessed 2023-10-20).

(82) Intratec. *Steam costs | Current and forecast*. https://www.intratec.us/products/water-utility-costs/commodity/steam-cost (accessed 2023-10-20).

(83) Yong Min Fen, C. Deep Decarbonisation of the Dutch Chemical Industry: A Marginal Abatement Cost Curve Analysis for LyondellBasell. **2020**, No. August.

(84) Statista. *Prices of electricity for non-household consumers in the Netherlands from 2008 to 2022*. https://www-statista-com.tudelft.idm.oclc.org/statistics/596254/electricity-non-household-price-netherlands/ (accessed 2023-10-20).

(85) Tan, M.; Ibarra-González, P.; Nikolic, I.; Ramírez Ramírez, A. Understanding the Level of Integration in Existing Chemical Clusters: Case Study in the Port of Rotterdam. *Circ. Econ. Sustain.* **2024**, No. submitted for review. https://doi.org/10.1007/s43615-024-00410-5.

(86) EEA. *Greenhouse gas emission intensity of electricity generation in Europe*. https://www.eea.europa.eu/en/analysis/indicators/greenhouse-gas-emission-intensity-of-1 (accessed 2025-05-16).

(87) statista. *Annual carbon dioxide emissions from energy use in Sweden from 1970 to 2023*. https://www.statista.com/statistics/449823/co2-emissions-sweden/ (accessed 2025-05-16).

(88) MCG Quantity. *ATO Depreciation - Effective Lives (2017-2018)*. https://www.mcgqs.com.au/ato-effective-lives-2017-2018-depreciation-rates/ (accessed 2024-08-29).

(89) EY. *Worldwide Capital and Fixed Assets Guide*; 2018. https://assets.ey.com/content/dam/ey-sites/ey-com/en_gl/topics/tax/guides/worldwide-capital-and-fixed-assets-guide-2018.pdf (accessed 2024-08-29).

(90) Manalal, J. T.; Pérez-Fortes, M.; Gonzalez, P. I.; Ramirez, A. R. Evaluation of Alternative Carbon Based Ethylene Production in a Petrochemical Cluster: Technology Screening & Value Chain Impact Assessment; 2023; pp 2453–2458. https://doi.org/10.1016/B978-0-443-15274-0.50390-5.
